# Effective Identification of Akt Interacting Proteins by Two-Step Chemical Crosslinking, Co-Immunoprecipitation and Mass Spectrometry

**DOI:** 10.1371/journal.pone.0061430

**Published:** 2013-04-17

**Authors:** Bill X. Huang, Hee-Yong Kim

**Affiliations:** Laboratory of Molecular Signaling, National Institute of Alcohol Abuse and Alcoholism, National Institutes of Health, Bethesda, Maryland, United States of America; Hungarian Academy of Sciences, Hungary

## Abstract

Akt is a critical protein for cell survival and known to interact with various proteins. However, Akt binding partners that modulate or regulate Akt activation have not been fully elucidated. Identification of Akt-interacting proteins has been customarily achieved by co-immunoprecipitation combined with western blot and/or MS analysis. An intrinsic problem of the method is loss of interacting proteins during procedures to remove non-specific proteins. Moreover, antibody contamination often interferes with the detection of less abundant proteins. Here, we developed a novel two-step chemical crosslinking strategy to overcome these problems which resulted in a dramatic improvement in identifying Akt interacting partners. Akt antibody was first immobilized on protein A/G beads using disuccinimidyl suberate and allowed to bind to cellular Akt along with its interacting proteins. Subsequently, dithiobis[succinimidylpropionate], a cleavable crosslinker, was introduced to produce stable complexes between Akt and binding partners prior to the SDS-PAGE and nanoLC-MS/MS analysis. This approach enabled identification of ten Akt partners from cell lysates containing as low as 1.5 mg proteins, including two new potential Akt interacting partners. None of these but one protein was detectable without crosslinking procedures. The present method provides a sensitive and effective tool to probe Akt-interacting proteins. This strategy should also prove useful for other protein interactions, particularly those involving less abundant or weakly associating partners.

## Introduction

Protein–protein interactions are essential for virtually all cellular processes. Disruption of protein interactions may alter protein function and biological events, thus contributing to the inception of diseases. Accordingly, identification of interacting proteins is of vital importance to advance our understanding of both biological [1] and disease processes, which may facilitate new therapeutic approaches [2], [3], [4].

The serine/threonine kinase Akt (also called protein kinase B) is a key signaling protein that controls cell proliferation, differentiation, apoptosis, glucose metabolism, transcription and cell migration [5]. The critical regulatory role in these diverse biological processes makes Akt an important drug target for the treatment of various diseases such as cancer and diabetes. Significant progress has been made to understand the molecular mechanisms of Akt activation. It is well established that Akt is recruited to the plasma membrane [6] and activated through phosphorylation of T308 and S473 by phosphoinositide-dependent protein kinase (PDK) 1 and mammalian target of rapamycin (mTOR) respectively [5], [7]. However, Akt binding partners (such as downstream substrates) and their interaction dynamics that modulate or regulate Akt activation in various physiological and pathological conditions have not been fully elucidated [8]. Therefore, an effective method to identify Akt-protein interaction is of great interest.

Co-immunoprecipitation (IP) combined with mass spectrometry (MS) has become the method of choice for identifying protein-protein interactions [9], [10], [11], [12]. In this experimental strategy, cells were lysed under non-denaturing conditions so that the protein interaction within the cell is not disrupted in the cell extract. When the target protein is captured (immunoprecipitated) with a specific antibody bound to protein agarose beads, interacting partners of this protein may also precipitate. After removing non-specifically bound proteins by repeated washing, co-immunoprecipitated interacting partners are typically eluted from the beads using Laemmli buffer, separated by SDS-polyacrylamide gel electrophoresis (SDS-PAGE), digested in-gel using proteases, and identified by tandem MS (MS/MS).

The most common problem encountered in a co-IP experiment is the loss of interacting partners during the wash process which is critical for the success of the technique [13], [14]. Ideally, washing should be sufficiently vigorous to maximally remove non-specifically bound proteins, while retaining co-precipitated interacting proteins on the beads. Fewer washes or use of less stringent buffers tend to identify more binding partners, but also significantly increase the number of non-specifically interacting proteins. Too extensive wash or use of a strong buffer containing high proportion of detergents and salts can decrease non-specific binding, but likely disrupts the association of proteins, resulting in inevitable loss of the interacting proteins, particularly low abundant or weakly bound partners. Optimization of the wash procedure therefore remains challenging in co-IP experiments. A co-IP/MS experiment for many proteins such as Akt normally requires a large amount of protein extract (greater than 10 mg proteins extracted typically from >10^8^ cells) just to detect abundant interacting partners [15], [16]. Another common problem in the co-IP approach is the antibody contamination during elution [17]. The co-elution of antibody often interferes with subsequent analysis of interacting proteins that are present at a low abundance.

Chemical crosslinking can be used to capture stable or transient protein-protein interactions into covalent complexes [18], [19], [20]. Taking advantage of this feature, we developed in the present study a novel two-step crosslinking strategy that overcomes the intrinsic co-IP problems with an aim to improve the identification of Akt interacting partners. Combined with mass spectrometric analysis, the crosslinking approach allowed us to identify ten Akt-binding partners from the cell lysates containing as low as 1.5 mg proteins (typically from ∼10^7^ cells), all of which but one protein were undetectable without the crosslinking procedures. Among those, two proteins were newly identified as potential Akt interacting partners. The interacting proteins for which specific antibodies are available were validated by western blot analyses, and their dynamic change during activation was further evaluated.

## Materials and Methods

### Reagents

Succinimidyl suberate (DSS), dithiobis[succinimidylpropionate] (DSP), protein A/G plus agarose, 100 X protease inhibitor cocktail without EDTA, ECL western blotting substrates, and BCA protein assay reagents, were obtained from Thermo Scientific. PBS (pH 7.4, 795 mg/L Na_2_HPO_4_, 144 mg/L KH_2_PO_4_, 9000 mg/mL NaCl, without calcium and magnesium) and MOPS SDS running buffer were purchased from Invitrogen. Tris-HCl (pH 7.4), rabbit serum, anti-rabbit IgG peroxidase secondary antibody, Igepal CA-60 (NP-40), and Laemmli buffer (2% SDS, 10% glycerol, 0.002% bromophenol blue, 125 mM Tris HCl) were purchased from Sigma. Rabbit anti-Akt antibody and anti-ERK1/2 antibody were purchased from Cell Signaling. Rabbit anti-EBP1 and mouse anti-Nudc antibodies were purchased from Abcam. Mouse anti-GSK antibody was obtained from Transduction.

### Cell Culture and IGF Stimulation

Neuro 2A (mouse neuroblastoma) cells purchased from American Type Culture Collection (ATCC, Manassas, VA) were grown at 37°C in Dulbecco’s modified Eagle’s medium (DMEM) supplemented with 5% fetal bovine serum (FBS) in a humidified atmosphere of 5% CO_2_. When confluent, cells were starved overnight in serum-free DMEM then stimulated with or without IGF-1 (10 ng/mL, PeproTech) for 10 min. After rinsing twice with cold PBS, cells were lysed in PBS buffer containing 1% NP-40 and protease inhibitors on ice for 45 min with occasional vortexes. After centrifugation at 15,000 g for 10 min, proteins were quantified by the Bradford protein assay, using bovine serum albumin as the standard..

### Two-step on-bead Chemical Crosslinking and Immunoprecipitation

Co-IP procedures were performed at 4°C unless otherwise indicated, using a Pierce spin column which can be capped and plugged with a bottom plug for incubation or unplugged to remove the supernatant by centrifugation at 1000 g for 1 minute. The binding of Akt antibody to protein A/G agarose was performed with the protocol described in Pierce crosslink immunoprecipitation kits with slight modification. Protein A/G agarose slurry (20 µl) was washed twice with 200 µl PBS buffer, and incubated with 100 µl Akt antibody prepared in PBS (10 µl Akt antibody +85 µl H_2_O+5 µl 20X PBS) at 25°C for 30 min on a mixer. In parallel, 100 µl of rabbit serum or anti-rabbit IgG peroxidase secondary antibody with same concentration of IgG as that of Akt antibody (0.38 µg/mL) was similarly prepared as the negative control. The supernatant was discard and the beads were washed three times with 300 µl PBS, followed by incubated with 50 µl DSS solution (2.5 µl 20 X PBS+38.5 µl H_2_O+2.5 mM DSS in DMSO) at 25°C for 45–60 min on a mixer. After removing the supernatant the beads were washed three times with 50 µl 100 mM glycine (pH 2.8), twice with 300 µl PBS buffer containing 1% NP-40, then once with 300 µl PBS. The antibody-cross-linked beads were incubated overnight at 4°C with 600 µl lysate of Neuro 2A cells which were pre-cleared with control agarose resin (Pierce) for 2 h on a shaker. The incubation continued after adding 20 µl 50 mM DSP in DMSO (final concentration of DSP = 1.6 mM) for 2 h. The DSP-crosslinking was quenched by adding 30 µl 1 M Tris-HCl pH 7.4 (30 min). After removing supernatant (flow-through) and washing with 600 µl washing buffer (25 mM Tris, 150 mM NaCl, 1 mM EDTA, 1% NP-40, 5% glycerol, pH 7.4) five times, the immunoprecipitates were eluted with 40 µl 2X Laemmli buffer at 100°C for 10 min. The cap of the spin column was loose to avoid overpressure and leakage from the bottom when boiling. The eluting complex was subjected to SDS-PAGE separation for MS analysis or western blotting. For the traditional co-IP experiment without using DSS crosslinking, the protein A/G agarose was incubated 10 µl Akt antibody at 25°C for 1 h on a mixer, followed by incubation with 600 µl pre-cleared lysate overnight. The immunoprecipitated products were washed with the washing buffer five times and eluted with 2X Laemmli buffer at 100°C for 10 min.

### SDS-PAGE and In-gel Digestion

Samples were loaded onto a 10% Bis-Tris gels (Invitrogen). Electrophoresis was carried out at a constant voltage of 50 V using MOPS SDS running buffer for approximate 25 min. The proteins were visualized with Coomassie blue (SimplyBlue SafeStain, Invitrogen). Entire gel were diced into small pieces (1–2 mm), distained with 25 mM NH_4_HCO_3_ in 50% ACN, dried by vacuum centrifugation, and subjected to in gel reduction and alkylation with 10 mM DTT and 55 mM iodoacetamide respectively. After sequential washing with 25 mM NH_4_HCO_3_, 25 mM NH_4_HCO_3_/50%ACN, and 100% ACN, gel pieces were dried and rehydrated with 12.5 ng/mL trypsin (Promega, Madison, WI) solution in 25 mM ammonium bicarbonate on ice for 30 min. The digestion was continued at 37°C overnight. The tryptic peptides were extracted with 5% formic acid/50% ACN, concentrated with vacuum centrifugation, and desalted using C-18 ziptip (Millipore).

### Nano-HPLC MS/MS Analysis

Nano-LC-ESI-MS/MS was performed on an LTQ-Orbitrap XL mass spectrometer (Thermo Scientific) equipped with an Eksigent nanoLC 1D system. The mobile phases consisted of 0.1% formic acid (solvent A) and 0.1% formic acid in 95% ACN (solvent B). Peptides were loaded onto a C18 trap column (Zorbax 5×0.3 mm, Agilent) and separated by a 15 cm IntegraFrit column (ProteoPep™, New Objective) at a flow rate of 300 nL/min with a gradient of solvent B changing from 5 to 40% over 150 min or 300 min. LC eluent was sprayed into the MS instrument with a glass emitter tip (PicoTip, New Objective) using a spray voltage of 2.0 kV in positive-ion mode. Full scan spectra from m/z 300 to 2000 at resolution of 60,000 were acquired in the Orbitrap. Ten data-dependent MS/MS spectra of most intense ions were acquired in the LTQ-XL ion trap using CID with a normalized energy of 35. Dynamic exclusion for the already fragmented precursor ions was used with the following parameters: exclusion time 180 s, repeat count 1, repeat duration 30 s, exclusion mass width 10 ppm, and exclusion size 500. Singly charged species were excluded from fragmentation.

### Western Blot Analysis

Samples were electrophoresed in 4–12% Bis-Tris gels at 100 V using MOPS SDS running buffer. Proteins were electrophoretically transferred to a PVDF membrane at 100 V for 1.5 hr. The membrane was blocked with 5% milk in TBS containing 0.1% Tween 20 (TBS-T) at room temperature for 1 h. Blots were incubated with primary antibody (at 1/1000 ratio) at 4°C overnight, washed three times with TBS-T, then incubated with peroxidase-conjugated secondary antibody for 1 hour at room temperature. After washing three times with TBS-T, blots were incubated with ECL detection reagent for 5 min, and imaged with a Kodak Gel Logic 440 Imaging. Band intensity was quantitated using Kodak 1D imaging analysis software.

### Protein Identification

The acquired MS/MS data were searched against the NCBInr mouse database (NCBInr 2011.09.02, 145,913 sequences) with Mascot (v2.3.2, Matrix Science) using Mascot Distiller (2.3.2.0) as the data input filter to generate peak lists. Alternatively, Bioworks (v.3.3.1, Thermo Scientific) was used as the search engine. Search parameters were set as follows: enzyme, trypsin; precursor ion mass tolerance, 10 ppm; fragment ion mass tolerance, 0.8 Da; maximum missed cleavages allowed 2; carbamidomethyl of cysteine residues for fixed modification; oxidation of methionine and addition of 145.0198 Da on lysine (CAMthiopropanoyl modification) for variable modification. The criteria used to filter results included 1% false positive threshold and expect value of less than 0.05 for significant peptide matches. The expect score was calculated using the homology threshold or the significance threshold as per a standard Mascot protein family report.

## Results and Discussion

### A Two-step on-bead Chemical Crosslinking Strategy for Co-immunoprecipitation

**Figure 1:**
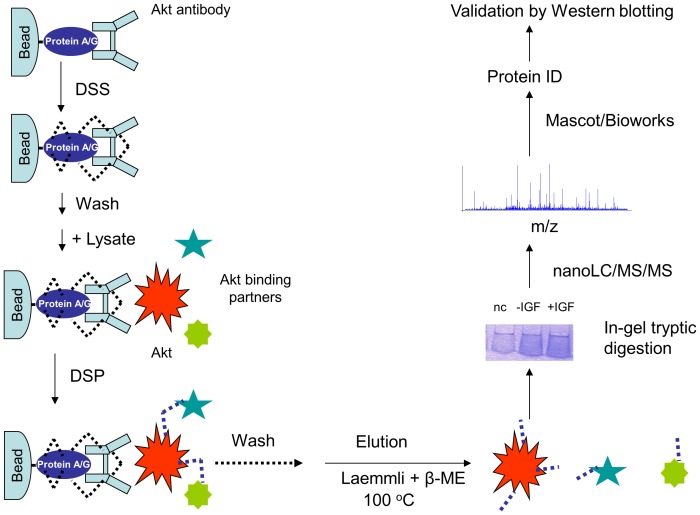
Schematic diagram of a two-step crosslinking approach for identification of Akt binding partners. Akt antibody was immobilized on protein A/G beads via DSS (succinimidyl suberate) cross-linking. Following the incubation of the cell lysate with the immobilized Akt antibody, DSP (dithiobis[succinimidylpropionate]), a cleavable crosslinker, was used to produce stable complexes between Akt and its binding partners. The co-immunoprecipitated complexes were eluted with Laemmli buffer containing 5% β-mercaptoethanol. The liberated proteins were subjected to SDS-PAGE, tryptic digestion, and MS-based analysis followed by western blot validation.

**Figure 2:**
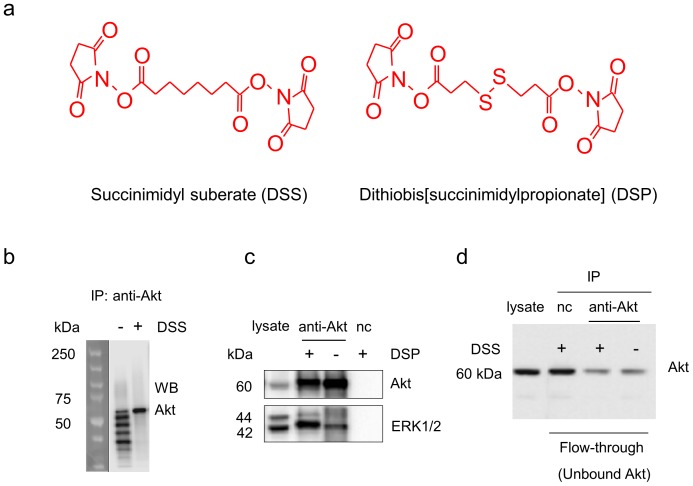
Western blot analysis of Akt and ERK1/2 monitored during co-IP procedures. a. Chemical structures of the crosslinkers used in the study. b. Antibody contamination was eliminated by DSS-crosslinking of Akt antibody to protein A/G agarose beads. c. ERK1/2 co-immunoprecipitation with Akt was significantly improved by DSP-crosslinking. d. DSS-crosslinking did not alter antibody-antigen binding. nc, negative control using IgG instead of Akt antibody at a similar protein level.

To overcome the dissociation of the interacting proteins as well as the antibody contamination during IP processes, we designed a sequential two-step crosslinking approach (Fig. 1). Two homo-bifunctional lysine specific cross-linkers were chosen (Fig. 2a); 1) non-cleavable disuccinimidyl suberate (DSS) to immobilize Akt antibody on protein A/G beads permanently, and 2) cleavable dithiobis[succinimidylpropionate] (DSP) to produce stable complexes between Akt and its binding partners. The crosslinkers can interact with amine groups from two lysine residues provided they are spatially located within ∼12 Å [21], thus crosslinking the interacting proteins via covalent bonds. In the first step where the antibody is crosslinked to the protein A/G beads by DSS (Fig. 1), a reaction time of 45–60 min (at room temperature) was used in an attempt to ensure maximum modification of reactive primary amine groups in the antibody IgG as well as protein A/G. Such inactivation of the amine groups can minimize the irrelevant capture of the background proteins onto the antibody surface or protein beads in the subsequent step of DSP-crosslinking between Akt and its interacting partners. Alternatively, dimethyl pimelimidate (DMP), an imidoester crosslinker that also reacts with primary amines, can be used for immobilizing the antibody to protein beads to avoid antibody contamination [22]. In this case, an additional buffer with higher pH values (e.g. triethanolamine or sodium borate, pH 8.5) is needed for optimal crosslinking activity of DMP [22]. The DSS-crosslinking was quenched and the excessive crosslinker and uncross-linked antibody was removed by washing with glycine solution. Subsequently, the beads with immobilized anti-Akt antibody were incubated with Neuro 2A cell lysate, and Akt and its binding partners were crosslinked with the cleavable crosslinker DSP. After extensive wash, the co-IP products were eluted with Laemmli buffer containing 5% β-mercaptoethanol that cleaves the disulfide bond in the DSP structure, and thus releasing Akt and its interacting partners. The liberated proteins were subjected to SDS-PAGE/in-gel tryptic digestion and nanoLC-ESI-MS/MS analysis.

The effectiveness of the strategy was evaluated by western blotting after co-IP experiments were performed with or without the crosslinking procedures (Fig. 2b–d). As expected, without DSS-crosslinking, heavy and light chains as well as fragments of IgG were detected in the IP product. The use of DSS completely eliminated the antibody contamination (Fig. 2b), indicating that Akt antibody was immobilized successfully on the protein A/G beads via DSS-crosslinking. Subsequent DSP-crosslinking significantly improved the detection of Akt-interacting proteins (2.5±0.2 fold increase normalized to Akt level), as demonstrated in Fig. 2c for extracellular regulated kinase-1 and -2 (ERK1/2), a known Akt-binding partner [23]. The data indicated that the dissociation of ERK1/2 from Akt during the wash process was prevented by DSP-crosslinking.

The antibody-antigen binding relies on specific structural recognition. The Y-shaped antibodies contains a paratope at the tip of the “Y” that is specific to one particular epitope on the antigen, allowing two structures to bind to each other. The activity of an antibody depends on the tip structure, or antigen binding site. A major concern regarding the immobilization of an antibody to protein beads is the possible alteration of the tip structure by crosslinking, masking the paratope that could adversely affect the binding to antigen and thus lowering the efficiency of the immunoprecipitation. We addressed the issue by evaluating Akt in the flow-through fraction (i.e, unbound Akt) after incubating lysates with the immobilized antibody. Unbound Akt detected in the flow-through fraction was comparable with and without DSS crosslinking (Fig. 2d, third and fourth lanes), indicating that immobilization using DSS at the concentration employed in this study did not alter the antigen-binding capability of the Akt antibody. Although thousands of antibodies share a general structure, tip structures are slightly different. Consequently, DSS-crosslinking may affect some antibodies more than others, possibly compromising the antibody-antigen interaction. In this regard, selecting a proper antibody is important for the crosslinking/IP approach.

### Analysis of Co-immunoprecipitated Proteins by Tandem Mass Spectrometry

**Figure 3:**
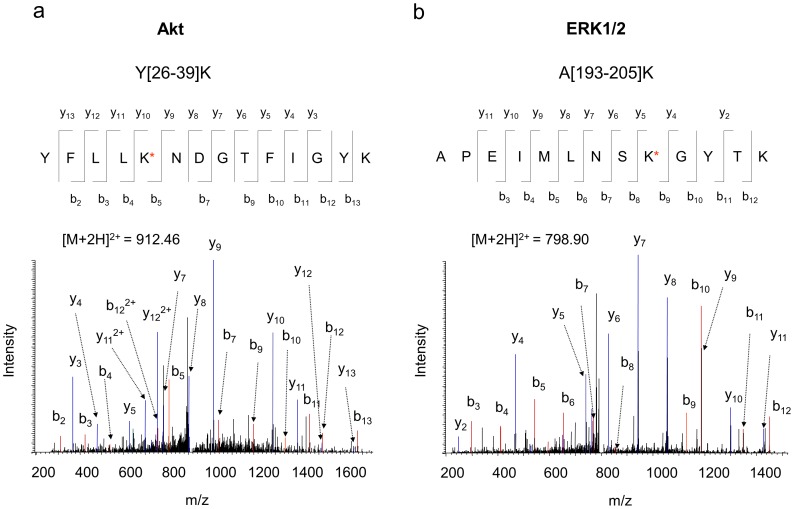
Representative MS/MS spectra of Akt and ERK1/2 peptides obtained from the co-IP complex. MS/MS analysis of the doubly charged ion at m/z 912.46 or 798.90 originated from the peptide segment Y[26]–[39]K of Akt (a), or A[193–205]K of ERK1/2 (b), respectively. The sequence of the peptide was assigned with a single letter abbreviation based on the fragment ions observed for the peptide segments. N-terminal b ions and C-terminal y ions resulting from the amide bond cleavage are labeled. * denotes the addition of 145.0198 Dalton due DSP-crosslinking.

Mass spectrometry has been a powerful tool to screen and identify new interacting proteins from the co-IP products, while western blot analysis can only be used to verify known or predicted interacting partners. After testing the proof-of-concept of the on-bead crosslinking by western blotting, we performed nanoLC-ESI-MS/MS analysis for the co-immunoprecipitated proteins that were digested in gel as described in the experimental section. Proteins were identified based on the MS/MS spectra of the peptides. Endogenous Akt, the bait protein used for the pull-down, was detected with 45% coverage of amino acid residues. In addition to the protein identity, tandem MS can reveal the exact crosslinking sites [24]. In the case of the crosslinking with DSP, either an end-capping (one side crosslinked with a lysine and the other side is hydrolyzed) or a crosslinking between two lysine residues, results in the lysine modification with an increase of 145.0198 Da (+C_5_H_7_NO_2_S) in mass after cleavage of the S-S bond (Fig. 2a). Such DSP-modifications can be readily identified from the acquired MS/MS data using Mascot software. For example, Fig. 3a shows a representative MS/MS spectrum of a peptide with mass of 1822.901 Da, reconstituted from doubly charged ion of m/z 912.46. The MS/MS data revealed that the peptide was originated from Y[26]–[39]K of Akt1 (gi/6753034), with lysine 30 modified by DSP. Note that once a lysine residue is modified, it is no longer cleavable by trypsin. Consistent with the western blot data shown in Fig. 2, an Akt binding partner ERK1/2, was identified with 19% sequence coverage in the protein complex pulled down by Akt. The MS/MS data of A[193–205]K revealed that K201 of ERK1/2 was involved in the DSP-modification (Fig. 3b). These modified residues may have resulted from the end-capping or internal crosslinking within the same protein. However, it is also possible that the DSP-modified lysine residues such as K30 of Akt and K201 of ERK1/2 were involved in the formation of the stable covalent complexes between interacting proteins.

### Identification of Interacting Partners of Akt in Neuro 2A Cells

An important issue in the MS-based co-IP approach is how to distinguish the bona fide interacting proteins from a large pool of non-specifically bound proteins. Because of the great improvement in detection capability of mass spectrometry, up to hundreds of proteins could be detected in a co-IP sample even after the most stringent purification processes [15], [25]. However, most identified proteins are non-specifically bound proteins. These background proteins often include abundant proteins such as actin, tubulin and heat shock proteins, even though an immunodepletion process can reduce these proteins considerably. In addition, some proteins may bind non-specifically to the protein A/G beads or antibody. To distinguish these irrelevant background proteins, we devised a negative control using serum IgG or a secondary antibody with the same concentration as that of Akt antibody used in the study. Because identical amount of IgG was immobilized to A/G beads by DSS, the negative control provided a similar affinity matrix to which background proteins bound. To test whether the DSP-crosslinking procedure leads to the identification of more background proteins, we examined another negative control without DSP-crosslinking. The identified protein list was comparable to the case using DSP, indicating that the DSP-crosslinking does not add more background proteins, presumably due to the fact that the preceding DSS-crosslinking to immobilize the antibody on the beads masked most reactive sites (primary amine groups) available.

To filter out the non-specific contaminants, we adapted stringent criteria in a manner similar to that reported previously [26]. First, we compared the protein identification results between the sample and the negative control with the help of Bioworks software. Common proteins including actin, elongation factor 1, tubulin, and Hsp90 were identified in both samples, and thus were considered background proteins, even though they have been reported as Akt binding partners [16], [27], [28]. Secondly, the proteins that were not reproduced in three independent experiments were filtered out. Using this approach, we identified ten Akt binding proteins from the lysate of Neuro 2A cells containing only 1.5 mg proteins (Tables S1, S2, S3). These proteins include ERK1/2, ErbB3 binding protein 1 (EBP1, also called proliferation-associated protein 2G4), alpha-actinin, valosin-containing protein (VCP), protein phosphatase 1 (PP-1), glycogen synthase kinase (GSK)-3β, 14-3-3, prohibitin-2, nuclear distribution gene C (Nudc, also named nuclear migration gene C) and transcription factor II-I (TFII-I) (Table 1). All these proteins were detected from the Akt-specific pull-down complexes, but not from the negative control where IgG or a secondary antibody was used instead of Akt antibody. Employing the conventional approach with neither DSS- nor DSP-crosslinking, none of these proteins were detected from the same lysate containing similar protein levels. When the Akt antibody was fixed on the beads via DSS but without DSP-crosslinking, only ERK1/2 was identified. Even then, ERK1/2 was detected at a sequence coverage much lower (5% from two distinct peptides) than the case with DSP-crosslinking (19% from six distinct peptides), despite a similar level of Akt detected (∼45% sequence coverage). The MS result was consistent with western blot analysis that showed a reduced level of ERK1/2 compared to the case with DSP-crosslinking (Fig. 2c). Considering that other nine interactors were not detected without the use of the crosslinking procedures, it is speculated that the ERK1/2-Akt interaction is relatively stronger or the ERK1/2 level is high in Neuro 2A cells. These results clearly demonstrate the advantage of the two-step crosslinking strategy in co-IP experiments, particularly in search for specific binding partners by mass spectrometry.

**Table 1:** Akt binding partners identified from Neuro 2A cells using a two-step crosslinking-co-IP/MS approach.

Protein name#	MW (kDa)	Accession number	Peptides identified by MS/MS search by Mascot		Sequence coverage
			sequence	Ion score/expect p	
EBP1^a^	42	116283229	T[200–211]KA[264–271]RF[321–332]RI[333–344]K	45/0.002439/0.009841/0.01446/0.0026	11%
ERK1/2^b^	42	6754632	G[14]–[22]RF[76–89]RI[163–170]RV[171–189]RE[343–351]RA[193–205]K^c^	43/0.00271/5.3e–0544/0.005258/9.7e–0535/0.0214.3^d^/2.1e–05	19%
α-actinin	105	11230802	L[302–311]RV[735–746]R	45/0.005791/1.7e–07	2%
VCP	90	55217	G[240–251]KE[366–377]RW[454–465]R	37/0.02527/0.0554/0.001	4%
PP-1	39	471976	Y[114–122]RI[169–187]R	52/0.003637/0.022	8%
GSK-3β^a^	47	9790077	I[384–405]RL[320–328]R^c^	126/8.2e–122.5^d^/3.6e–03	7.5%
14-3-3 ζ/δ	28	6756041	S[28]–[41]RV[61–74]R	52/0.001582/3.5e–06	11%
Prohibitin-2	33	126723336	I[55–71]R	53/0.00021	3%
Nudc^b^	38	6754910	L[194–205]RG[214–228]K	55/0.001942/0.0013	8%
TFII I	113	124001574	S[515–523]RR[540–555]R	54/0.002555/0.00022	2%

# None of these proteins were detected without using DSP-crosslinking, with the exception of ERK1/2. Without DSP-crosslinking ERK1/2 was identified by only two distinct peptides (G[14]–[22]R and A[193–201]K) with 5% sequence coverage.

a Proteins found to be more associated with active Akt than inactive Akt by western blot analysis.

b Association with Akt found to be independent of the activation status of Akt revealed by western blot analysis.

c peptides identified by Bioworks (3.1).

d XC score.

### Identification of New Akt Interacting Partners

Among the ten Akt binding partners summarized in Table 1, the first eight proteins have been reported previously as Akt interacting partners and their biological significance have been documented. In brief, it has been shown that EBP1 interacts with activated nuclear Akt to prevent DNA fragmentation and suppress apoptosis [29]. ERK1/2 co-exists with Akt in a multimolecular complex containing PDK1 and Rsk, and inhibition of ERK1/2 activity induced up-regulation of Akt promoting cell survival in MK-PT cells [23]. VCP is required for Akt-mediated cell survival in MCF-7 breast cancer cells [30]. Prohibitin-2 is a transcription repressor which binds to MyoD, a myogenic regulatory factor [31]. By competitive binding, Akt reduces the prohibitin binding to MyoD, and thus mediates the transcription activity of MyoD and stimulates muscle differentiation. PP-1 is a major phosphatase that dephosphorylates Akt at Thr450 and modulates Akt functions in regulating gene expression, cell survival and differentiation [32]. 14-3-3 coordinates with Akt to inhibit the function of proapoptotic proteins including BAD and FOXO thus promote cell survival [33], [34], [35]. GSK-3β is a downstream substrate of Akt. Akt phosphorylates and inactivates GSK-3β, allowing activation of signaling pathways that are blocked by GSK to proceed [36]. Alpha-actinin is a modulator for Akt membrane translocation and phosphorylation [37].

**Figure 4:**
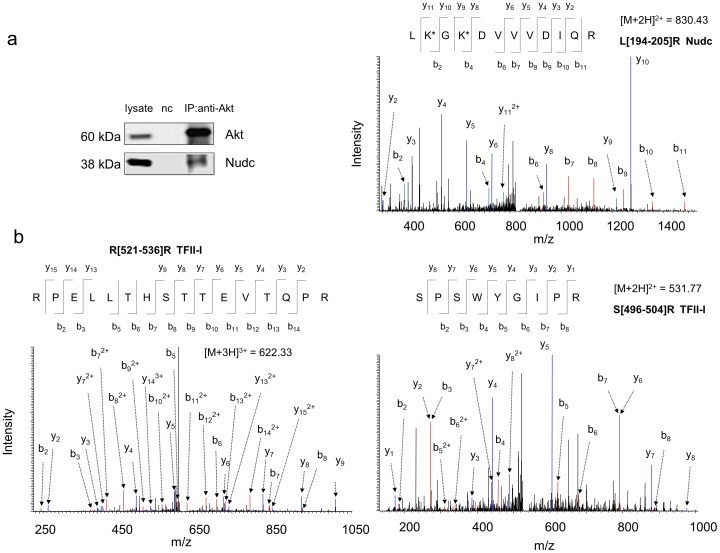
Identification of Nudc and TFII-I as potential Akt binding partners. Representative MS/MS spectra of Nudc (a) and TFII-I (b) peptides identified from the co-IP products. The sequence of peptides was assigned with a single letter abbreviation based on the fragment ions observed for the peptide segments. N-terminal b ions and C-terminal y ions resulting from the amide bond cleavage are labeled. * denotes the addition of 145.0198 Dalton due DSP-crosslinking. Western blotting indicated that Nudc co-immunoprecipitated with Akt in Neuro 2A cells (a). nc, negative control using IgG instead of Akt antibody at a similar protein level.

In addition to these known Akt binding partners, the current approach enabled the identification of Nudc and TFII-I as potential Akt binding partners in Neuro 2A cells (Fig. 4). Both western blot analysis and MS-based identification consistently indicated the interaction between Nudc and Akt (Fig. 4a). The Akt interaction with TFII-I was identified by the MS approach only (Fig. 4b); possibly due to the low level of TFII-1 in Neuro 2A cells to be detected by the currently available antibodies. Nudc is a nuclear movement protein that plays a role in various biological processes including mitosis, cytokinesis and cell proliferation. It has been demonstrated that Nudc is phosphorylated at S275 and S327, by polo-like kinase 1 (PLK-1), a serine/threonine protein kinase [38]. TFII-1 is an important multifunction transcriptional regulator that is also involved in various signal transduction pathways. It has been shown that TFII-I is activated through either tyrosine phosphorylation by Bruton’s tyrosine kinase [39] or serine phosphorylation at S371 and S743 by cGMP-dependent protein kinase I [40]. Interestingly, the primary sequences of both Nudc and TFII-I contain an Akt-phosphorylation consensus motif (RxRxxS/T), namely RARQET^79^ and RGREFS^703^ respectively. These proteins also encode two sequence variants, WNRLVT^260^/DPRQKS^299^ (Nudc), MLRDQS^146^/TKRLKS^307^ (TFII-I), where the first arginine at the conserved motif is lacking but can also be phosphorylated by Akt at the threonine or serine in some cases [16]. These structural information suggest that Nudc and TFII-I are possible targets of Akt. It should be emphasized that our results at this stage only suggest the potential Akt binding partners. Reversed IP can be employed to confirm the interaction, however, its success largely depends on individual antibodies since their binding to the antigen proteins often mask the interacting sites of the proteins. Unfortunately, currently available anti-Nudc or TFII-I antibodies were not proper for IP experiments. Further biochemical and molecular biological approaches such as over-expressing or silencing interacting proteins are required to validate the observed interaction and their functional significance.

### Dynamic Change in Akt-protein Interactions Associated with Akt Activation

**Figure 5:**
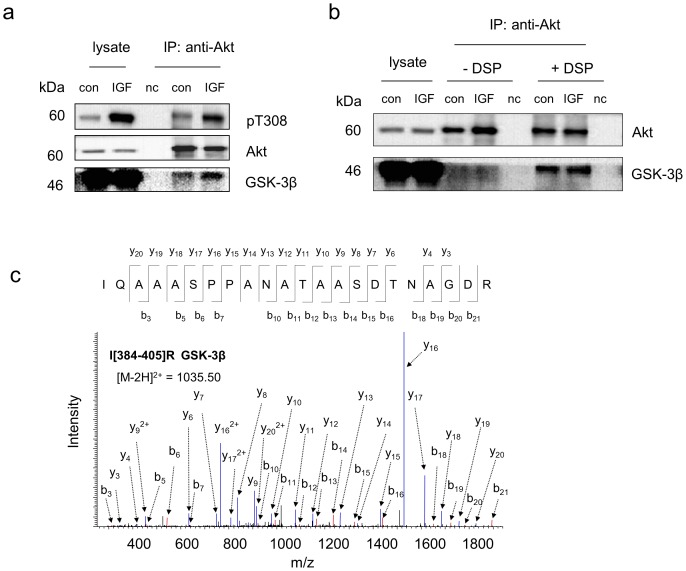
IGF-dependent interaction of Akt with GSK-3β revealed by the crosslinking/co-IP approach. a, Western blot analysis indicating that active Akt is associated with GSK-3β more than inactive Akt. The active Akt status was indicated by phosphorylation of T308. b, Western blot analysis of the co-IP complex with or without DSP-crosslinking following the incubation of the cell lysate with Akt antibody immobilized on the beads by DSS. GSK-3β was detected in either control or IGF-stimulated samples only when DSP-crosslinking was used. c, Representative MS/MS spectrum of a peptide corresponding to GSK-3β identified only with DSP-crosslinking approach. The sequence of the peptide was assigned with a single letter abbreviation based on the fragment ions observed for the peptide segments. N-terminal b ions and C-terminal y ions resulting from the amide bond cleavage are labeled. Con, control sample without IGF stimulation. IGF, IGF-stimulated samples obtained by treating Neuro 2A cells with IGF at 10 ng/mL for 10 min. nc, negative control using IgG instead of Akt antibody at a similar protein level. * denotes the addition of 145.0198 Dalton due DSP-crosslinking.

**Figure 6:**
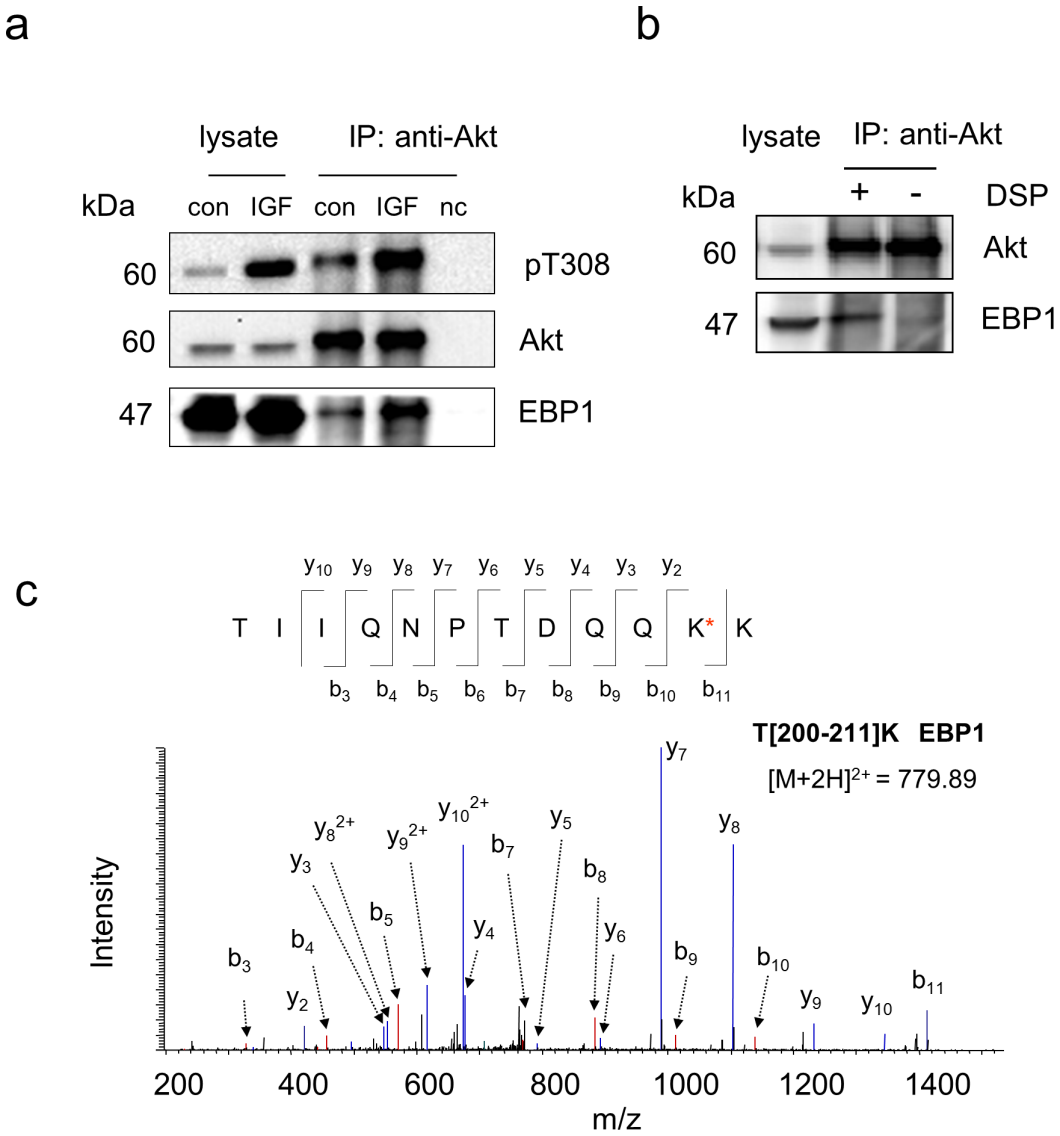
IGF-dependent interaction of Akt with EBP1. a, Western blot analysis indicating that active Akt is associated with EBP1 more than inactive Akt. The active Akt status was indicated by phosphorylation of T308. b, Western blot analysis of the co-IP complex with or without DSP-crosslinking following the incubation of the IGF-stimulated cell lysate with Akt antibody immobilized on the beads by DSS. EBP1 was negligibly detected in the IP product without the use of DSP-crosslinking. c, Representative MS/MS spectrum of a peptide corresponding to EBP1 identified only with DSP-crosslinking approach. The sequence of the peptide was assigned with a single letter abbreviation based on the fragment ions observed for the peptide segments. N-terminal b ions and C-terminal y ions resulting from the amide bond cleavage are labeled. Con, control sample without IGF stimulation. IGF, IGF-stimulated samples obtained by treating Neuro 2A cells with IGF at 10 ng/mL for 10 min. nc, negative control using IgG instead of Akt antibody at a similar protein level. * denotes the addition of 145.0198 Dalton due DSP-crosslinking.

Since the activation status of Akt may affect Akt-protein interactions, we also compared Akt binding partners under basal and an insulin-like growth factor (IGF)-simulated conditions in Neuro 2A cells. All Akt-interacting proteins identified under an inactive state were detected by MS when Akt was activated by 10-min IGF stimulation (Table 1). However, western blot analysis indicated that the interactions of two individual proteins with Akt changed upon Akt activation. As shown in Fig. 5a and 6a, GSK-3β and EBP1 were more associated with active Akt than inactive Akt (1.8±0.2 and 2.4±0.4 fold increases normalized to Akt level, respectively based on two biological replicates). It is worthwhile to note that the crosslinking strategy allowed monitoring these changes, as GSK-3β or EBP1 pulled down by Akt was detected only when DSP-crosslinking was used (Fig. 5b,c and Fig. 6b,c respectively). The observed IGF-dependent association of EBP1 is in agreement with the notion that EBP1 interacts mainly with activated Akt in PC-12 cells [29].

**Figure 7:**
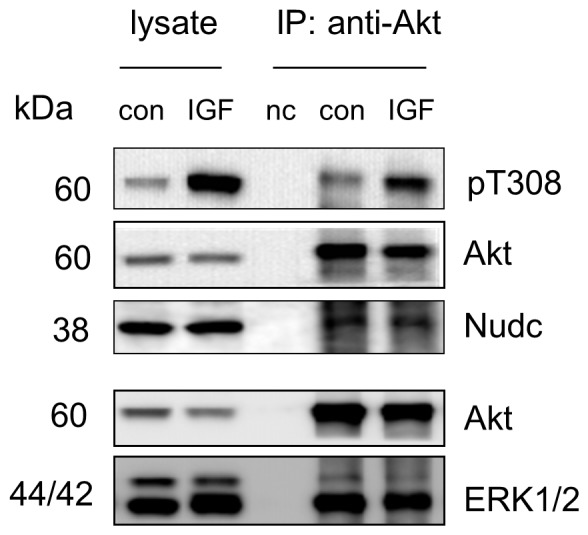
IGF-independent interaction of Akt with ERK1/2 or Nudc revealed by western blot analysis. The active Akt status was indicated by phosphorylation of T308. Con, control sample without IGF stimulation. IGF-stimulated samples were obtained by treating Neuro 2A cells with IGF at 10 ng/mL for 10 min. nc, negative control using IgG instead of Akt antibody at a similar protein level.

Unlike these cases, the binding of ERK1/2 was independent of the activation states of Akt in Neuro 2A cells (0.98±0.4 fold change normalized to Akt level, based on two biological replicates), as shown in Fig. 7. Our data is similar to the previous report that Akt-ERK1/2 interaction is unaltered after Akt activation by epidermal growth factor (EGF) in MK-PT cells [23]. The Akt association with Nudc, a newly identified Akt-interacting protein, was not affected by Akt activation, either, at least after 10 min IGF stimulation (0.97±0.1 fold change normalized to Akt, Fig. 7). To gain insight for the significance of protein interaction dynamics in Akt functions and downstream events, the stoichiometry of these interactions should be further investigated in a more quantitative manner under various conditions (e.g, at different time points of IGF stimulation) [17], [41]. For this purpose, quantitative mass spectrometric techniques such as stable isotope labeling by amino acids in cell culture (SILAC) [42], [43] or *in vitro* isobaric tag for relative and absolute quantitation (iTRAQ) [44] can be readily adapted to our crosslinking-co-IP approach.

### Detection of Low Abundance Binding Partners

It is intriguing that the present crosslinking approach allowed for the detection of GSK-3β, a known Akt substrate with low abundance, in cell lysate containing as low as 1.5 mg proteins (∼1×10^7^ cells). Using a classic co-IP coupled with MS-based proteomics approach Vandermoere et al. have identified valosin-containing protein and actin as new substrates for Akt in breast cancer cells [16], [30]. In their study, nine other previously reported Akt binding proteins, including alpha-actinin, IkB kinase beta, mortalin (GRP 70), tubulin beta, cytokeratin 8, proliferating cell nuclear antigen, 14-3-3 sigma and heat shock protein 27, were found to be co-immunoprecipitated with Akt from lysate containing 15 mg of total proteins. However, the inability to detect some of the well-established partners with low abundance such as GSK-3β indicates an intrinsic limitation of detecting low abundance proteins in conventional co-IP/MS techniques [15], [16]. To increase the probability for detecting co-IP proteins, a large-scale preparation of cell lysates was required. Typically 10–20 mg proteins from the lysate was sufficient for abundant proteins [15]. However, the use of more than 10 g lysate proteins has also been reported for less abundant membrane proteins such as nicotinic receptor-associated proteins [45]. Although the detection of interacting partners relies on the type of interaction (i.e., strong or weak) and the cellular copy numbers of those proteins, our data indicated that inevitable loss of interacting proteins during the removal of nonspecific background proteins, is also a significant factor contributing to the limitation. This problem was overcome by employing the sequential crosslinking approach described in this study, as exemplified by the detection of GSK-3β and EBP1, which were not detected without the crosslinking procedures (Table 1, Fig. 5 and 6). In this regard, the crosslinking approach should be valuable when obtaining large amount of samples is troublesome.

### Conclusions

We have developed a novel two-step crosslinking strategy for the identification of Akt-interacting proteins by co-IP coupled to tandem mass spectrometry. Once the interacting proteins are identified by mass spectrometry, confirmation and dynamic comparison can be readily performed using targeted western blot analyses as long as specific antibodies are available. Due to the ability to overcome the loss of interacting proteins and antibody contamination, this approach with sequential crosslinking dramatically improved the identification of Akt binding partners with respect to conventional methods. This approach can provide a sensitive tool for readily probing Akt-protein interactions under both physiological and pathological conditions. The simple yet effective method should also be applicable to other protein-protein interactions, particularly for identifying low abundant or weakly bound interacting proteins, or when the quantity of biological samples is limited.

## Supporting Information

Table S1 Proteins identified in a negative control sample.
(DOCX)

Table S2 Proteins identified in the co-IP products from a non-IGF-stimulated sample.
(DOCX)

Table S3 Proteins identified in the co-IP products from an IGF-stimulated sample.
(DOCX)
