# Supplementary material for: Effective Identification of Akt Interacting Proteins by Two-Step Chemical Crosslinking, Co-Immunoprecipitation and Mass Spectrometry
Source: PLoS One. 2013 Apr 17;8(4):e61430. doi: 10.1371/journal.pone.0061430 (PMC3629208; doi:10.1371/journal.pone.0061430)
Supplement: Table S1 — Proteins identified in a negative control sample. (DOCX) [file pone.0061430.s001.docx]

Table S1. Proteins identified in a negative control sample

| Family | Member | Accession | Score | Mass (kDa) | # of Sig. sequences | Description |
| --- | --- | --- | --- | --- | --- | --- |
| 1 | 1 | gi\|7106439 | 888 | 50095 | 16 | tubulin beta-5 chain |
| 1 | 2 | gi\|13542680 | 745 | 50239 | 15 | tubulin, beta 2C |
| 1 | 3 | gi\|12963615 | 611 | 50842 | 12 | tubulin beta-3 chain |
| 1 | 4 | gi\|27754056 | 381 | 50514 | 8 | tubulin beta-6 chain |
| 1 | 5 | gi\|225480 | 274 | 51016 | 4 | tubulin Mbeta 1 |
| 2 | 1 | gi\|74220592 | 845 | 70876 | 21 | unnamed protein product |
| 2 | 2 | gi\|12835845 | 374 | 72492 | 10 | unnamed protein product |
| 3 | 1 | gi\|84662730 | 608 | 67573 | 17 | far upstream element-binding protein 1 |
| 3 | 2 | gi\|148706277 | 566 | 79634 | 15 | mCG140911, isoform CRA_a |
| 3 | 3 | gi\|159163538 | 327 | 9772 | 6 | Chain A, solution structure of Kh domain in fuse binding protein 1 |
| 3 | 4 | gi\|148676563 | 68 | 61694 | 2 | mCG130458 |
| 4 | 1 | gi\|40556608 | 538 | 83571 | 21 | heat shock protein HSP 90-beta |
| 4 | 2 | gi\|194033 | 250 | 39864 | 8 | heat-shock protein hsp86, partial |
| 4 | 3 | gi\|14714615 | 167 | 92717 | 4 | heat shock protein 90, beta (Grp94), member 1 |
| 5 | 1 | gi\|6755901 | 513 | 50788 | 17 | tubulin alpha-1A chain |
| 5 | 2 | gi\|148667971 | 261 | 28130 | 8 | tubulin, alpha 4, isoform CRA_b |
| 6 | 1 | gi\|31980648 | 376 | 56265 | 9 | ATP synthase subunit beta, mitochondrial precursor |
| 7 | 1 | gi\|6679937 | 366 | 36072 | 10 | glyceraldehyde-3-phosphate dehydrogenase |
| 8 | 1 | gi\|6754994 | 364 | 37987 | 10 | poly(rC)-binding protein 1 |
| 8 | 2 | gi\|1360003 | 284 | 37203 | 7 | nuclear poly(C)-binding protein, splicevariant E |
| 8 | 3 | gi\|148699884 | 278 | 36171 | 5 | poly(rC) binding protein 3, isoform CRA_b |
| 9 | 1 | gi\|407261700 | 329 | 45501 | 11 | elongation factor 1-alpha 1-like isoform 3 |
| 10 | 1 | gi\|16716569 | 303 | 26802 | 1 | protease, serine, 1 precursor |
| 11 | 1 | gi\|6679439 | 248 | 18131 | 8 | peptidyl-prolyl cis-trans isomerase A |
| 12 | 1 | gi\|74204605 | 219 | 73731 | 6 | unnamed protein product |
| 13 | 1 | gi\|16303309 | 213 | 61971 | 4 | type II keratin 5 |
| 13 | 2 | gi\|126116585 | 188 | 66079 | 3 | keratin, type II cytoskeletal 1 |
| 13 | 3 | gi\|54607171 | 175 | 59641 | 3 | keratin, type II cytoskeletal 6A |
| 13 | 4 | gi\|148672085 | 143 | 38347 | 2 | mCG144996 |
| 13 | 5 | gi\|148672069 | 103 | 59516 | 2 | cDNA sequence BC031593 |
| 14 | 1 | gi\|488513 | 212 | 68661 | 4 | EWS |
| 15 | 1 | gi\|6681219 | 204 | 62296 | 6 | dihydropyrimidinase-related protein 3 isoform 2 |
| 16 | 1 | gi\|171948782 | 201 | 32944 | 5 | laminin receptor |
| 17 | 1 | gi\|14318588 | 193 | 68547 | 9 | Sf1 protein |
| 18 | 1 | gi\|26341628 | 190 | 60439 | 6 | unnamed protein product |
| 19 | 1 | gi\|809561 | 182 | 41335 | 6 | actin |
| 20 | 1 | gi\|473912 | 181 | 51190 | 5 | phosphoprotein |
| 21 | 1 | gi\|18204423 | 180 | 64751 | 6 | Picalm protein |
| 22 | 1 | gi\|6680748 | 179 | 59830 | 7 | ATP synthase subunit alpha, mitochondrial precursor |
| 23 | 1 | gi\|5020213 | 177 | 37360 | 7 | mitotic checkpoint protein BUB3 |
| 24 | 1 | gi\|19526912 | 176 | 41801 | 6 | hsc70-interacting protein |
| 25 | 1 | gi\|18606229 | 169 | 52397 | 5 | 5830457O10Rik protein |
| 26 | 1 | gi\|387397 | 165 | 57978 | 5 | epidermal keratin subunit I, partial |
| 26 | 2 | gi\|148670626 | 93 | 55010 | 6 | mCG144006 |
| 26 | 3 | gi\|154090941 | 90 | 50444 | 4 | keratin, type I cytoskeletal 42 |
| 26 | 4 | gi\|11559579 | 87 | 52420 | 5 | keratin intermediate filament 16a |
| 26 | 5 | gi\|6680606 | 74 | 44515 | 5 | keratin, type I cytoskeletal 19 |
| 26 | 6 | gi\|387399 | 59 | 10712 | 2 | epidermal keratin type I, partial |
| 27 | 1 | gi\|60502437 | 149 | 49026 | 2 | protein disulfide isomerase associated 6 |
| 28 | 1 | gi\|6753320 | 149 | 61162 | 3 | T-complex protein 1 subunit gamma |
| 29 | 1 | gi\|26346937 | 137 | 33111 | 2 | unnamed protein product |
| 30 | 1 | gi\|10946928 | 136 | 49454 | 4 | heterogeneous nuclear ribonucleoprotein H |
| 31 | 1 | gi\|46485130 | 134 | 86156 | 2 | TPA_exp: keratin Kb40 |
| 32 | 1 | gi\|18079339 | 132 | 86151 | 4 | aconitate hydratase, mitochondrial precursor |
| 33 | 1 | gi\|86476054 | 129 | 68248 | 4 | VGF nerve growth factor inducible precursor |
| 34 | 1 | gi\|398168 | 126 | 71447 | 2 | keratin 2 epidermis |
| 35 | 1 | gi\|3550456 | 123 | 96520 | 5 | Alix |
| 36 | 1 | gi\|6679399 | 122 | 29741 | 2 | paired mesoderm homeobox protein 2A |
| 37 | 1 | gi\|4504445 | 120 | 34289 | 1 | heterogeneous nuclear ribonucleoprotein A1 isoform a |
| 38 | 1 | gi\|468546 | 120 | 57753 | 5 | CCT (chaperonin containing TCP-1) beta subunit |
| 39 | 1 | gi\|13529524 | 119 | 71527 | 2 | Ubap2 protein, partial |
| 40 | 1 | gi\|220494 | 117 | 65548 | 2 | MSEC66 |
| 41 | 1 | gi\|53000 | 110 | 271556 | 3 | microtubule-associated protein 1B |
| 42 | 1 | gi\|21704096 | 105 | 44918 | 2 | TAR DNA-binding protein 43 isoform 1 |
| 43 | 1 | gi\|6755372 | 103 | 26828 | 3 | 40S ribosomal protein S3 |
| 44 | 1 | gi\|4502201 | 103 | 20741 | 3 | ADP-ribosylation factor 1 |
| 45 | 1 | gi\|3329496 | 103 | 88635 | 2 | heterogenous nuclear ribonucleoprotein U |
| 56 | 1 | gi\|26345686 | 97 | 57344 | 2 | unnamed protein product |
| 47 | 1 | gi\|17978023 | 95 | 227414 | 2 | nonmuscle heavy chain myosin II-A |
| 48 | 1 | gi\|74207626 | 89 | 35483 | 2 | unnamed protein product |
| 49 | 1 | gi\|201725 | 89 | 57952 | 5 | t complex polypeptide 1 |
| 50 | 1 | gi\|5295992 | 88 | 60044 | 3 | chaperonin containing TCP-1 theta subunit |
| 51 | 1 | gi\|51455 | 86 | 61074 | 3 | heat shock protein 65 |
| 52 | 1 | gi\|483918 | 85 | 42846 | 2 | glutamate-ammonia ligase |
| 53 | 1 | gi\|26339248 | 83 | 117320 | 2 | unnamed protein product |
| 54 | 1 | gi\|8895708 | 83 | 43281 | 2 | DAZ-associated protein 1 |
| 55 | 1 | gi\|41946089 | 81 | 175327 | 1 | Eif4g1 protein |
| 56 | 1 | gi\|22094075 | 81 | 33138 | 2 | ADP/ATP translocase 2 |
| 57 | 1 | gi\|50409 | 81 | 77895 | 2 | unnamed protein product |
| 58 | 1 | gi\|48428375 | 80 | 176092 | 1 | RecName: Full=eukaryotic translation initiation factor 4 gamma 3 |
| 59 | 1 | gi\|1335839 | 77 | 24935 | 2 | thymopoietin zeta |
| 60 | 1 | gi\|3097244 | 77 | 16462 | 2 | ribosomal protein S14 |
| 61 | 1 | gi\|74198651 | 76 | 47582 | 1 | unnamed protein product |
| 62 | 1 | gi\|124028629 | 75 | 37437 | 2 | RecName: Full=heterogeneous nuclear ribonucleoproteins A2/B1 |
| 63 | 1 | gi\|13278582 | 74 | 56779 | 2 | Clint1 protein |
| 64 | 1 | gi\|12848426 | 73 | 28154 | 2 | unnamed protein product |
| 65 | 1 | gi\|19223941 | 72 | 34807 | 1 | mitotic phosphoprotein 44 |
| 66 | 1 | gi\|74212334 | 71 | 70824 | 2 | unnamed protein product |
| 67 | 1 | gi\|6756051 | 70 | 49719 | 3 | zinc finger protein 207 isoform 4 |
| 68 | 1 | gi\|26325842 | 68 | 56446 | 2 | unnamed protein product |
| 69 | 1 | gi\|74152131 | 68 | 59410 | 2 | unnamed protein product |
| 70 | 1 | gi\|973182 | 67 | 58899 | 1 | CD98 heavy chain |
| 71 | 1 | gi\|51092303 | 66 | 27255 | 1 | Try10-like trypsinogen precursor |
| 72 | 1 | gi\|6754976 | 66 | 22390 | 2 | peroxiredoxin-1 |
| 73 | 1 | gi\|6671702 | 66 | 60042 | 1 | T-complex protein 1 subunit epsilon |
| 74 | 1 | gi\|74200884 | 65 | 43660 | 2 | unnamed protein product |
| 75 | 1 | gi\|74151643 | 65 | 60159 | 3 | unnamed protein product |
| 76 | 1 | gi\|12850047 | 65 | 64775 | 1 | unnamed protein product |
| 77 | 1 | gi\|12843046 | 65 | 27074 | 1 | unnamed protein product |
| 78 | 1 | gi\|23956214 | 62 | 75508 | 2 | splicing factor, proline- and glutamine-rich |
| 79 | 1 | gi\|13324684 | 61 | 10009 | 1 | protein transport protein Sec61 subunit beta |
| 80 | 1 | gi\|74141990 | 61 | 60654 | 1 | unnamed protein product |
| 81 | 1 | gi\|1110559 | 60 | 27846 | 1 | WBP-2=Yes-associated protein WW domain-binding protein type 2 |
| 82 | 1 | gi\|22094989 | 60 | 39980 | 1 | mitochondrial import inner membrane translocase subunit TIM50 precursor |
| 83 | 1 | gi\|60360562 | 60 | 191457 | 1 | mKIAA4178 protein |
| 84 | 1 | gi\|398048 | 59 | 17951 | 1 | ribosomal protein L12 |
| 85 | 1 | gi\|460317 | 58 | 58598 | 1 | chaperonin |
| 86 | 1 | gi\|6425103 | 56 | 87418 | 3 | SEC23B protein |
| 87 | 1 | gi\|124517678 | 56 | 185956 | 1 | tyrosine-protein phosphatase non-receptor type 23 |
| 88 | 1 | gi\|6014493 | 55 | 67336 | 1 | PLIC-2 |
| 89 | 1 | gi\|130636 | 55 | 91628 | 1 | RecName: Full=probable pol polyprotein |
| 90 | 1 | gi\|6755893 | 55 | 26941 | 1 | trypsin 4 precursor |
| 91 | 1 | gi\|4590328 | 54 | 141523 | 1 | valyl-tRNA synthetase |
| 92 | 1 | gi\|31981560 | 54 | 46889 | 3 | stromal membrane-associated protein 2 |
| 92 | 2 | gi\|148682456 | 47 | 28799 | 2 | stromal membrane-associated protein 1, isoform CRA_a |
| 93 | 1 | gi\|6755963 | 53 | 30851 | 1 | voltage-dependent anion-selective channel protein 1 |
| 94 | 1 | gi\|4519256 | 53 | 56550 | 1 | 4F2/CD98 light chain |
| 95 | 1 | gi\|124249109 | 53 | 242852 | 1 | AT-rich interactive domain-containing protein 1A |
| 96 | 1 | gi\|4506413 | 53 | 21316 | 1 | ras-related protein Rap-1A |
| 97 | 1 | gi\|13385168 | 52 | 29634 | 1 | cytochrome b-c1 complex subunit Rieske, mitochondrial |
| 98 | 1 | gi\|6754222 | 51 | 30926 | 1 | heterogeneous nuclear ribonucleoprotein A/B isoform 2 |
| 99 | 1 | gi\|1167510 | 51 | 14281 | 1 | TI-225 |
| 100 | 1 | gi\|12230430 | 48 | 41772 | 1 | RecName: Full=poly(rC)-binding protein 4; AltName: Full=Alpha-CP4 |
| 101 | 1 | gi\|13543181 | 47 | 40955 | 1 | Hnrpm protein, partial |
| 102 | 1 | gi\|200397 | 47 | 57042 | 2 | phospholipase C-alpha |
| 103 | 1 | gi\|7242156 | 47 | 25120 | 1 | acyl-protein thioesterase 2 |
| 104 | 1 | gi\|50814 | 45 | 44692 | 2 | unnamed protein product |
| 105 | 1 | gi\|387422 | 44 | 36044 | 2 | malate dehydrogenase |
| 106 | 1 | gi\|148672614 | 43 | 73422 | 2 | RAN GTPase activating protein 1, isoform CRA_b |
| 107 | 1 | gi\|19527026 | 43 | 35312 | 1 | leucine-rich repeat-containing protein 59 |
| 108 | 1 | gi\|16741234 | 43 | 39849 | 1 | Gorasp2 protein |
| 109 | 1 | gi\|6671569 | 43 | 34366 | 1 | 60S acidic ribosomal protein P0 |
| 110 | 1 | gi\|30089704 | 42 | 13912 | 1 | histone H2B type 1-K |
| 111 | 1 | gi\|23346437 | 42 | 44384 | 1 | splicing factor 3B subunit 4 |
| 112 | 1 | gi\|85701680 | 41 | 63319 | 1 | keratin, type II cytoskeletal 2 oral |
| 113 | 1 | gi\|17512472 | 40 | 29518 | 2 | Penta-EF hand domain containing 1 |
| 114 | 1 | gi\|28175768 | 39 | 88240 | 1 | Atxnl2 protein, partial |
| 115 | 1 | gi\|19526818 | 39 | 40063 | 1 | phosphate carrier protein, mitochondrial precursor |
| 116 | 1 | gi\|6678439 | 39 | 26871 | 1 | anionic trypsin-2 precursor |
| 117 | 1 | gi\|26326219 | 39 | 88531 | 1 | unnamed protein product |
| 118 | 1 | gi\|6679261 | 38 | 43888 | 1 | pyruvate dehydrogenase E1 component subunit alpha, somatic form |
| 119 | 1 | gi\|91171 | 38 | 50850 | 1 | peripherin (clone 3u) - mouse (fragment) |
| 120 | 1 | gi\|51304 | 37 | 14653 | 1 | unnamed protein product |
| 121 | 1 | gi\|13399310 | 37 | 18904 | 1 | 40S ribosomal protein S10 |
| 122 | 1 | gi\|21898577 | 36 | 127126 | 1 | nogo-A |
| 123 | 1 | gi\|12841240 | 36 | 28325 | 2 | unnamed protein product |
| 124 | 1 | gi\|40549395 | 35 | 248353 | 1 | ankyrin repeat domain-containing protein 17 isoform b |
| 125 | 1 | gi\|37360414 | 35 | 74678 | 1 | mKIAA1499 protein |
| 125 | 1 | gi\|20072723 | 35 | 22433 | 1 | Rab5c protein, partial |
| 127 | 1 | gi\|10946934 | 34 | 33597 | 1 | THAP domain-containing protein 11 |
| 128 | 1 | gi\|6678349 | 34 | 43703 | 1 | nucleolysin TIAR |
| 129 | 1 | gi\|902342 | 33 | 35986 | 1 | human homolog is GPI-anchored protein |
| 130 | 1 | gi\|21704138 | 33 | 31300 | 1 | arf-GAP domain and FG repeat-containing protein 2 isoform 1 |
| 131 | 1 | gi\|12836885 | 33 | 123917 | 1 | nonsense mRNA reducing factor 1 NORF1 |
| 132 | 1 | gi\|18043455 | 33 | 30523 | 1 | aspartate dehydrogenase domain containing |
| 133 | 1 | gi\|21668096 | 33 | 54790 | 1 | lactation elevated 1 |
| 134 | 1 | gi\|7949055 | 33 | 22438 | 1 | hippocalcin-like protein 1 |
| 135 | 1 | gi\|26332465 | 32 | 37516 | 1 | unnamed protein product |
| 136 | 1 | gi\|53912 | 31 | 17781 | 1 | ribosomal protein L7 |
| 137 | 1 | gi\|4633656 | 31 | 66313 | 1 | phenylalanyl tRNA synthetase beta subunit |
| 138 | 1 | gi\|50400412 | 31 | 139586 | 1 | RecName: Full=E3 ubiquitin-protein ligase DZIP3 |
| 139 | 1 | gi\|257467625 | 30 | 292361 | 1 | Fc fragment of IgG binding protein-like precursor |
| 140 | 1 | gi\|26344812 | 30 | 66899 | 1 | unnamed protein product |
| 141 | 1 | gi\|20982845 | 29 | 52870 | 1 | RNA-binding protein FUS |
| 142 | 1 | gi\|627837 | 27 | 425711 | 1 | All-1 protein +GTE form - mouse (fragment) |
| 143 | 1 | gi\|15341745 | 27 | 61681 | 1 | EH-domain containing 4 |
| 144 | 1 | gi\|12847471 | 26 | 76472 | 1 | unnamed protein product |
| 145 | 1 | gi\|148670518 | 26 | 223216 | 1 | talin 1, isoform CRA_a |
| 146 | 1 | gi\|9910124 | 24 | 10023 | 1 | protein AF1q |
| 147 | 1 | gi\|20178023 | 23 | 147823 | 1 | RecName: Full=periaxin |
| 148 | 1 | gi\|26327249 | 23 | 29841 | 1 | unnamed protein product |
| 149 | 1 | gi\|1816635 | 22 | 123826 | 1 | SRG3 |
| 150 | 1 | gi\|17511228 | 22 | 63535 | 1 | RecQ helicase protein-like 5 beta |
| 151 | 1 | gi\|70794816 | 21 | 47453 | 1 | uncharacterized protein LOC433182 |
| 152 | 1 | gi\|12855399 | 20 | 125528 | 1 | unnamed protein product |
