# Supplementary material for: Effective Identification of Akt Interacting Proteins by Two-Step Chemical Crosslinking, Co-Immunoprecipitation and Mass Spectrometry
Source: PLoS One. 2013 Apr 17;8(4):e61430. doi: 10.1371/journal.pone.0061430 (PMC3629208; doi:10.1371/journal.pone.0061430)
Supplement: Table S2 — Proteins identified in the co-IP products from a non-IGF-stimulated sample. (DOCX) [file pone.0061430.s002.docx]

Table S2. Proteins identified in the co-IP products from a non-IGF-stimulated sample

| Family | Member | Accession | Score | Mass (kDa) | # of Sig. sequences | Description |
| --- | --- | --- | --- | --- | --- | --- |
| 1 | 1 | gi\|407261700 | 1055 | 45501 | 21 | elongation factor 1-alpha 1-like isoform 3 |
| 1 | 2 | gi\|6681273 | 437 | 50764 | 12 | elongation factor 1-alpha 2 |
| 2 | 1 | gi\|6755901 | 992 | 50788 | 19 | tubulin alpha-1A chain |
| 2 | 2 | gi\|148667971 | 517 | 28130 | 12 | tubulin, alpha 4, isoform CRA_b |
| 2 | 3 | gi\|148700287 | 25 | 48732 | 2 | mCG1728 |
| 3 | 1 | gi\|74220592 | 962 | 70876 | 23 | unnamed protein product |
| 3 | 2 | gi\|12835845 | 702 | 72492 | 16 | unnamed protein product |
| 4 | 1 | gi\|7106439 | 854 | 50095 | 18 | tubulin beta-5 chain |
| 4 | 2 | gi\|12963615 | 654 | 50842 | 14 | tubulin beta-3 chain |
| 4 | 3 | gi\|13542680 | 647 | 50239 | 16 | tubulin, beta 2C |
| 4 | 4 | gi\|27754056 | 258 | 50514 | 6 | tubulin beta-6 chain |
| 5 | 1 | gi\|26348171 | 691 | 47056 | 14 | unnamed protein product |
| 5 | 2 | gi\|74140319 | 624 | 51077 | 12 | unnamed protein product |
| 6 | 1 | gi\|74204605 | 623 | 73731 | 15 | unnamed protein product |
| 7 | 1 | gi\|40556608 | 592 | 83571 | 18 | heat shock protein HSP 90-beta |
| 7 | 2 | gi\|6754254 | 412 | 85134 | 12 | heat shock protein HSP 90-alpha |
| 7 | 3 | gi\|14714615 | 164 | 92717 | 4 | heat shock protein 90, beta (Grp94), member 1 |
| 8 | 1 | gi\|6679439 | 539 | 18131 | 15 | peptidyl-prolyl cis-trans isomerase A |
| 9 | 1 | gi\|6681219 | 539 | 62296 | 10 | dihydropyrimidinase-related protein 3 isoform 2 |
| 9 | 2 | gi\|1915913 | 259 | 62531 | 4 | Ulip2 protein |
| 10 | 1 | gi\|6680674 | 507 | 56105 | 14 | RAC-beta serine/threonine-protein kinase (Akt2) |
| 10 | 2 | gi\|190883484 | 475 | 56078 | 14 | RAC-gamma serine/threonine-protein kinase (Akt3) |
| 10 | 3 | gi\|6753034 | 468 | 56071 | 18 | RAC-alpha serine/threonine-protein kinase (Akt1) |
| 11 | 1 | gi\|74213524 | 496 | 42066 | 11 | unnamed protein product |
| 11 | 2 | gi\|809561 | 451 | 41335 | 10 | actin |
| 12 | 1 | gi\|6679937 | 464 | 36072 | 13 | glyceraldehyde-3-phosphate dehydrogenase |
| 12 | 2 | gi\|55153885 | 455 | 36093 | 12 | Glyceraldehyde-3-phosphate dehydrogenase |
| 13 | 1 | gi\|6680748 | 353 | 59830 | 9 | ATP synthase subunit alpha, mitochondrial precursor |
| 14 | 1 | gi\|74220566 | 320 | 56221 | 7 | unnamed protein product |
| 15 | 1 | gi\|468546 | 308 | 57753 | 5 | CCT (chaperonin containing TCP-1) beta subunit |
| 16 | 1 | gi\|29436595 | 304 | 30023 | 6 | protein-L-isoaspartate (D-aspartate) O-methyltransferase 1, partial |
| 17 | 1 | gi\|387397 | 275 | 57978 | 5 | epidermal keratin subunit I, partial |
| 17 | 2 | gi\|7106335 | 151 | 48417 | 3 | keratin, type I cytoskeletal 17 |
| 17 | 3 | gi\|387399 | 146 | 10712 | 2 | epidermal keratin type I, partial |
| 17 | 4 | gi\|11559579 | 101 | 52420 | 4 | keratin intermediate filament 16a |
| 17 | 5 | gi\|154090941 | 81 | 50444 | 3 | keratin, type I cytoskeletal 42 |
| 18 | 1 | gi\|387422 | 238 | 36044 | 5 | malate dehydrogenase |
| 19 | 1 | gi\|31982332 | 232 | 42834 | 5 | glutamine synthetase |
| 20 | 1 | gi\|70794816 | 225 | 47453 | 7 | uncharacterized protein LOC433182 |
| 21 | 1 | gi\|26346937 | 217 | 33111 | 3 | unnamed protein product |
| 21 | 2 | gi\|22094075 | 181 | 33138 | 3 | ADP/ATP translocase 2 |
| 22 | 1 | gi\|26346713 | 216 | 60111 | 6 | unnamed protein product |
| 23 | 1 | gi\|4759034 | 206 | 49228 | 5 | eukaryotic peptide chain release factor subunit 1 |
| 24 | 1 | gi\|18079339 | 196 | 86151 | 7 | aconitate hydratase, mitochondrial precursor |
| 25 | 1 | gi\|12859782 | 177 | 66099 | 3 | unnamed protein product |
| 26 | 1 | gi\|6754994 | 177 | 37987 | 4 | poly(rC)-binding protein 1 |
| 26 | 2 | gi\|1360003 | 175 | 37203 | 4 | nuclear poly(C)-binding protein, splicevariant E |
| 27 | 1 | gi\|74224349 | 176 | 77218 | 4 | unnamed protein product |
| 28 | 1 | gi\|12846283 | 175 | 24521 | 5 | unnamed protein product |
| 29 | 1 | gi\|488513 | 169 | 68661 | 3 | EWS |
| 30 | 1 | gi\|60502437 | 166 | 49026 | 4 | protein disulfide isomerase associated 6 |
| 31 | 1 | gi\|84662730 | 165 | 67573 | 5 | far upstream element-binding protein 1 |
| 31 | 2 | gi\|159163538 | 128 | 9772 | 3 | chain A, solution structure Of Kh domain in fuse binding protein 1 |
| 32 | 1 | gi\|148672069 | 141 | 59516 | 2 | cDNA sequence BC031593 |
| 32 | 2 | gi\|148672085 | 120 | 38347 | 2 | mCG144996 |
| 32 | 3 | gi\|16303309 | 117 | 61971 | 3 | type II keratin 5 |
| 32 | 4 | gi\|293686 | 76 | 59756 | 2 | epidermal keratin subunit II |
| 33 | 1 | gi\|20809354 | 156 | 59422 | 5 | polypyrimidine tract binding protein 1 |
| 34 | 1 | gi\|21450129 | 155 | 45129 | 3 | acetyl-CoA acetyltransferase, mitochondrial precursor |
| 35 | 1 | gi\|2690302 | 140 | 47781 | 2 | aspartate aminotransferase precursor |
| 36 | 1 | gi\|257743039 | 140 | 40075 | 6 | L-lactate dehydrogenase A chain isoform 2 |
| 37 | 1 | gi\|124028629 | 140 | 37437 | 3 | RecName: Full=heterogeneous nuclear ribonucleoproteins A2/B1 |
| 38 | 1 | gi\|473912 | 140 | 51190 | 4 | phosphoprotein |
| 39 | 1 | gi\|33239431 | 134 | 56689 | 5 | protein RCC2 |
| 40 | 1 | gi\|26353794 | 133 | 57103 | 7 | unnamed protein product |
| 41 | 1 | gi\|116283229 | 132 | 41824 | 4 | proliferation-associated protein 2G4 (EBP1) |
| 42 | 1 | gi\|74214542 | 128 | 43278 | 3 | unnamed protein product |
| 43 | 1 | gi\|9790077 | 126 | 47194 | 1 | glycogen synthase kinase-3 beta |
| 44 | 1 | gi\|23271826 | 126 | 112497 | 2 | ubiquitin associated protein 2-like |
| 45 | 1 | gi\|26353116 | 121 | 51470 | 4 | unnamed protein product |
| 45 | 2 | gi\|148670393 | 68 | 37396 | 3 | mCG50680 |
| 46 | 1 | gi\|460317 | 117 | 58598 | 4 | chaperonin |
| 47 | 1 | gi\|226005 | 115 | 32846 | 2 | protein 40kD |
| 48 | 1 | gi\|31541939 | 112 | 35632 | 3 | chitinase domain-containing protein 1 isoform 2 precursor |
| 49 | 1 | gi\|74204678 | 112 | 96138 | 3 | unnamed protein product |
| 50 | 1 | gi\|21704096 | 111 | 44918 | 2 | TAR DNA-binding protein 43 isoform 1 |
| 51 | 1 | gi\|110625979 | 106 | 50371 | 4 | elongation factor 1-gamma |
| 52 | 1 | gi\|21703842 | 106 | 55727 | 3 | tRNA-splicing ligase RtcB homolog |
| 53 | 1 | gi\|6754632 | 105 | 41648 | 5 | mitogen-activated protein kinase 1 (ERK1) |
| 54 | 1 | gi\|6755372 | 104 | 26828 | 1 | 40S ribosomal protein S3 |
| 55 | 1 | gi\|74186692 | 100 | 88760 | 1 | unnamed protein product |
| 56 | 1 | gi\|37360114 | 100 | 148389 | 4 | mKIAA0829 protein |
| 57 | 1 | gi\|4590328 | 99 | 141523 | 2 | valyl-tRNA synthetase |
| 58 | 1 | gi\|26340966 | 99 | 70730 | 1 | unnamed protein product |
| 59 | 1 | gi\|11230802 | 98 | 105368 | 2 | alpha-actinin |
| 60 | 1 | gi\|21450325 | 97 | 22297 | 4 | flavin reductase (NADPH) |
| 61 | 1 | gi\|12851415 | 96 | 29963 | 2 | unnamed protein product |
| 62 | 1 | gi\|551295 | 96 | 58394 | 3 | pyruvate kinase M |
| 63 | 1 | gi\|74202572 | 96 | 50385 | 2 | unnamed protein product |
| 64 | 1 | gi\|3550456 | 96 | 96520 | 2 | Alix |
| 65 | 1 | gi\|6753324 | 94 | 58424 | 1 | T-complex protein 1 subunit zeta |
| 66 | 1 | gi\|6677775 | 94 | 14807 | 2 | 60S ribosomal protein L22 |
| 67 | 1 | gi\|6009521 | 92 | 99934 | 3 | p100 co-activator |
| 68 | 1 | gi\|6671664 | 90 | 67635 | 2 | calnexin precursor |
| 69 | 1 | gi\|6753086 | 87 | 35867 | 3 | DNA-(apurinic or apyrimidinic site) lyase |
| 70 | 1 | gi\|66267550 | 86 | 168127 | 2 | Eprs protein, partial |
| 71 | 1 | gi\|16716569 | 85 | 26802 | 1 | protease, serine, 1 precursor |
| 72 | 1 | gi\|29293809 | 85 | 120564 | 3 | ATP-citrate synthase isoform 2 |
| 73 | 1 | gi\|109157405 | 85 | 62800 | 2 | chain A, Crystal Structure Of Mouse Amf |
| 74 | 1 | gi\|6754976 | 85 | 22390 | 1 | peroxiredoxin-1 |
| 75 | 1 | gi\|387129 | 84 | 36625 | 1 | cytosolic malate dehydrogenase, partial |
| 76 | 1 | gi\|5020213 | 82 | 37360 | 3 | mitotic checkpoint protein BUB3 |
| 77 | 1 | gi\|6756041 | 82 | 27925 | 2 | 14-3-3 protein zeta/delta |
| 78 | 1 | gi\|247242 | 82 | 61111 | 2 | heat shock protein hsp60, hsp60=chaperonin |
| 79 | 1 | gi\|201725 | 80 | 57952 | 2 | t complex polypeptide 1 |
| 80 | 1 | gi\|545439 | 80 | 2112 | 1 | Erp61, GRP58=stress-inducible luminal endoplasmic reticulum protein |
| 81 | 1 | gi\|1083269 | 79 | 59890 | 3 | CW17R protein - mouse |
| 82 | 1 | gi\|124001574 | 77 | 112708 | 2 | general transcription factor II-I isoform 1 (TFII-I) |
| 83 | 1 | gi\|19526912 | 76 | 41801 | 1 | hsc70-interacting protein |
| 84 | 1 | gi\|6680229 | 76 | 24318 | 3 | high mobility group protein B2 |
| 85 | 1 | gi\|4502201 | 75 | 20741 | 2 | ADP-ribosylation factor 1 |
| 86 | 1 | gi\|74204146 | 74 | 34259 | 1 | unnamed protein product |
| 87 | 1 | gi\|3065925 | 74 | 28193 | 1 | 14-3-3 protein beta |
| 88 | 1 | gi\|26345686 | 71 | 57344 | 1 | unnamed protein product |
| 89 | 1 | gi\|33468931 | 70 | 61641 | 1 | serine--tRNA ligase, cytoplasmic isoform 1 |
| 90 | 1 | gi\|124487483 | 70 | 117495 | 1 | 2-oxoglutarate dehydrogenase-like, mitochondrial |
| 91 | 1 | gi\|26345208 | 70 | 64740 | 3 | unnamed protein product |
| 92 | 1 | gi\|148708840 | 68 | 15004 | 2 | mCG7881 |
| 93 | 1 | gi\|6754222 | 68 | 30926 | 1 | heterogeneous nuclear ribonucleoprotein A/B isoform 2 |
| 94 | 1 | gi\|1083440 | 66 | 54678 | 1 | octamer-binding protein NonO - mouse |
| 95 | 1 | gi\|6671539 | 65 | 39787 | 1 | fructose-bisphosphate aldolase A isoform 2 |
| 96 | 1 | gi\|55217 | 65 | 89936 | 3 | valosin-containing protein |
| 97 | 1 | gi\|12805413 | 64 | 31636 | 1 | Echs1 protein |
| 98 | 1 | gi\|555823 | 62 | 58283 | 2 | pendulin |
| 99 | 1 | gi\|47847498 | 62 | 155577 | 2 | mFLJ00279 protein |
| 100 | 1 | gi\|497775 | 61 | 55112 | 2 | fascin |
| 101 | 1 | gi\|1666689 | 60 | 221379 | 1 | alpha-NAC, muscle-specific form gp220 |
| 102 | 1 | gi\|251417 | 59 | 2455 | 1 | glutathione S-transferase {N terminus, Peak II pI 8.7} |
| 103 | 1 | gi\|22094123 | 58 | 120988 | 1 | transcription elongation factor SPT5 |
| 104 | 1 | gi\|6679108 | 58 | 32711 | 1 | nucleophosmin isoform 1 |
| 105 | 1 | gi\|6754910 | 58 | 38334 | 2 | nuclear migration protein nudc |
| 106 | 1 | gi\|130636 | 57 | 91628 | 1 | RecName: Full=probable pol polyprotein |
| 107 | 1 | gi\|6679803 | 57 | 11972 | 1 | peptidyl-prolyl cis-trans isomerase FKBP1A |
| 108 | 1 | gi\|13096978 | 57 | 72511 | 1 | Poly(A) binding protein, cytoplasmic 4 |
| 109 | 1 | gi\|28077013 | 57 | 47972 | 1 | stromal membrane-associated protein 1 |
| 110 | 1 | gi\|148696763 | 57 | 49906 | 1 | mCG1048875, isoform CRA_c |
| 111 | 1 | gi\|18606229 | 56 | 52397 | 2 | 5830457O10Rik protein |
| 112 | 1 | gi\|66571305 | 55 | 64410 | 1 | nuclear RNA export factor 3 |
| 113 | 1 | gi\|2253158 | 55 | 57969 | 1 | peripherin |
| 114 | 1 | gi\|12848861 | 55 | 31398 | 2 | unnamed protein product |
| 115 | 1 | gi\|398048 | 54 | 17951 | 1 | ribosomal protein L12 |
| 116 | 1 | gi\|6753320 | 54 | 61162 | 2 | T-complex protein 1 subunit gamma |
| 117 | 1 | gi\|126723336 | 53 | 33276 | 1 | prohibitin-2 |
| 118 | 1 | gi\|20149756 | 53 | 47095 | 1 | eukaryotic initiation factor 4A-III |
| 119 | 1 | gi\|199025 | 53 | 118230 | 2 | microtubule-associated protein 4 |
| 120 | 1 | gi\|6678359 | 52 | 68272 | 2 | transketolase |
| 121 | 1 | gi\|86476054 | 52 | 68248 | 1 | VGF nerve growth factor inducible precursor |
| 122 | 1 | gi\|23956214 | 51 | 75508 | 1 | splicing factor, proline- and glutamine-rich |
| 123 | 1 | gi\|6680345 | 50 | 43157 | 2 | isocitrate dehydrogenase [NAD] subunit gamma 1, mitochondrial precursor |
| 124 | 1 | gi\|2992164 | 50 | 64202 | 1 | eIF3 p66 |
| 125 | 1 | gi\|7242156 | 49 | 25120 | 1 | acyl-protein thioesterase 2 |
| 126 | 1 | gi\|7305075 | 48 | 51854 | 1 | ras GTPase-activating protein-binding protein 1 |
| 127 | 1 | gi\|387106 | 48 | 46488 | 1 | aspartate aminotransferase |
| 128 | 1 | gi\|26324732 | 48 | 59428 | 2 | unnamed protein product |
| 129 | 1 | gi\|19526818 | 48 | 40063 | 1 | phosphate carrier protein, mitochondrial precursor |
| 130 | 1 | gi\|9506571 | 48 | 36371 | 1 | eukaryotic translation initiation factor 2 subunit 1 |
| 131 | 1 | gi\|255958286 | 47 | 36474 | 2 | succinyl-CoA ligase [ADP/GDP-forming] subunit alpha, mitochondrial precursor |
| 132 | 1 | gi\|18204423 | 46 | 64751 | 1 | Picalm protein |
| 133 | 1 | gi\|13435747 | 45 | 23434 | 1 | Rho GDP dissociation inhibitor (GDI) alpha |
| 134 | 1 | gi\|471976 | 44 | 39211 | 2 | protein phosphatase 1 (PP-1) |
| 135 | 1 | gi\|46485130 | 42 | 86156 | 1 | TPA_exp: keratin Kb40 |
| 136 | 1 | gi\|25955503 | 42 | 120059 | 1 | Sec24 related gene family, member C (S. cerevisiae) |
| 137 | 1 | gi\|51092303 | 42 | 27255 | 1 | Try10-like trypsinogen precursor |
| 138 | 1 | gi\|124487457 | 42 | 86396 | 1 | MAP7 domain-containing protein 2 |
| 139 | 1 | gi\|14250204 | 42 | 101876 | 1 | methylenetetrahydrofolate dehydrogenase (NADP+ dependent) |
| 140 | 1 | gi\|398168 | 42 | 71447 | 1 | keratin 2 epidermis |
| 141 | 1 | gi\|3461880 | 41 | 61218 | 1 | guanine nucleotide regulatory protein |
| 142 | 1 | gi\|2745894 | 41 | 51414 | 1 | putative RNA helicase RCK |
| 143 | 1 | gi\|26336489 | 40 | 107713 | 1 | unnamed protein product |
| 144 | 1 | gi\|148685701 | 40 | 28018 | 1 | Tial1 cytotoxic granule-associated RNA binding protein-like 1 |
| 145 | 1 | gi\|26324430 | 40 | 49991 | 1 | unnamed protein product |
| 146 | 1 | gi\|26346400 | 37 | 57567 | 1 | unnamed protein product |
| 147 | 1 | gi\|45219736 | 36 | 65320 | 1 | Abce1 protein, partial |
| 148 | 1 | gi\|124486783 | 36 | 136863 | 1 | bromodomain and PHD finger-containing protein 3 |
| 149 | 1 | gi\|6756051 | 36 | 49719 | 1 | zinc finger protein 207 isoform 4 |
| 150 | 1 | gi\|1184661 | 35 | 56948 | 1 | vacuolar adenosine triphosphatase subunit B |
| 151 | 1 | gi\|1184659 | 35 | 68567 | 1 | vacuolar adenosine triphosphatase subunit A |
| 152 | 1 | gi\|885932 | 33 | 21963 | 1 | peroxidase |
| 153 | 1 | gi\|11464733 | 33 | 46664 | 1 | pseudouridine synthase 1 |
| 155 | 1 | gi\|26332465 | 32 | 37516 | 1 | unnamed protein product |
| 156 | 1 | gi\|20809759 | 32 | 53815 | 1 | Tulp1 protein |
| 157 | 1 | gi\|1705525 | 32 | 83145 | 1 | RecName: Full=DNA replication licensing factor MCM5 |
| 158 | 1 | gi\|82900057 | 32 | 20660 | 1 | PREDICTED: nucleoside diphosphate kinase B-like |
| 159 | 1 | gi\|3851614 | 32 | 59249 | 1 | succinate dehydrogenase Fp subunit |
| 160 | 1 | gi\|12845960 | 31 | 16163 | 1 | unnamed protein product |
| 161 | 1 | gi\|30424663 | 31 | 38774 | 1 | putative transferase CAF17 homolog, mitochondrial isoform 1 precursor |
| 162 | 1 | gi\|13435984 | 30 | 56239 | 1 | serine hydroxymethyltransferase 2 (mitochondrial) |
| 163 | 1 | gi\|169790797 | 30 | 287488 | 1 | Fc fragment of IgG binding protein precursor |
| 164 | 1 | gi\|74180983 | 30 | 274980 | 1 | unnamed protein product |
| 165 | 1 | gi\|26326779 | 30 | 135415 | 1 | unnamed protein product |
| 166 | 1 | gi\|20982845 | 30 | 52870 | 1 | RNA-binding protein FUS |

Proteins highlighted were not found in the negative control samples and reproduced in three independent experiments. These proteins were identified as Akt binding partners.
