# Supplementary material for: Effective Identification of Akt Interacting Proteins by Two-Step Chemical Crosslinking, Co-Immunoprecipitation and Mass Spectrometry
Source: PLoS One. 2013 Apr 17;8(4):e61430. doi: 10.1371/journal.pone.0061430 (PMC3629208; doi:10.1371/journal.pone.0061430)
Supplement: Table S3 — Proteins identified in the co-IP products from an IGF-stimulated sample. (DOCX) [file pone.0061430.s003.docx]

Table S3. Proteins identified in the co-IP products from an IGF-stimulated sample

| Family | Member | Accession | Score | Mass (kDa) | # of Sig. sequences | Description |
| --- | --- | --- | --- | --- | --- | --- |
| 1 | 1 | gi\|407261700 | 1206 | 45501 | 22 | elongation factor 1-alpha 1-like isoform 3 |
| 1 | 2 | gi\|6681273 | 611 | 50764 | 13 | elongation factor 1-alpha 2 |
| 2 | 1 | gi\|74198858 | 1135 | 71080 | 28 | unnamed protein product |
| 2 | 2 | gi\|12835845 | 702 | 72492 | 17 | unnamed protein product |
| 3 | 1 | gi\|7106439 | 1066 | 50095 | 23 | tubulin beta-5 chain |
| 3 | 2 | gi\|12963615 | 924 | 50842 | 19 | tubulin beta-3 chain |
| 3 | 3 | gi\|13542680 | 904 | 50239 | 19 | tubulin, beta 2C |
| 3 | 4 | gi\|21746161 | 867 | 50377 | 16 | tubulin beta-2B chain |
| 3 | 5 | gi\|27754056 | 521 | 50514 | 11 | tubulin beta-6 chain |
| 4 | 1 | gi\|6755901 | 1046 | 50788 | 17 | tubulin alpha-1A chain |
| 4 | 2 | gi\|148667971 | 529 | 28130 | 11 | tubulin, alpha 4, isoform CRA_b |
| 4 | 3 | gi\|148700287 | 52 | 48732 | 2 | mCG1728 |
| 5 | 1 | gi\|74204605 | 882 | 73731 | 19 | unnamed protein product |
| 6 | 1 | gi\|40556608 | 824 | 83571 | 24 | heat shock protein HSP 90-beta |
| 6 | 2 | gi\|6754254 | 466 | 85134 | 16 | heat shock protein HSP 90-alpha |
| 6 | 3 | gi\|14714615 | 152 | 92717 | 4 | heat shock protein 90, beta (Grp94), member 1 |
| 7 | 1 | gi\|6681219 | 632 | 62296 | 11 | dihydropyrimidinase-related protein 3 isoform 2 |
| 7 | 2 | gi\|148678069 | 602 | 73748 | 11 | dihydropyrimidinase-like 3, isoform CRA_a |
| 7 | 3 | gi\|1915913 | 291 | 62531 | 4 | Ulip2 protein |
| 8 | 1 | gi\|74202196 | 570 | 50988 | 18 | unnamed protein product |
| 8 | 2 | gi\|74140319 | 470 | 51077 | 13 | unnamed protein product |
| 9 | 1 | gi\|6679937 | 550 | 36072 | 14 | glyceraldehyde-3-phosphate dehydrogenase |
| 10 | 1 | gi\|6679439 | 521 | 18131 | 12 | peptidyl-prolyl cis-trans isomerase A |
| 11 | 1 | gi\|74151565 | 457 | 56294 | 11 | unnamed protein product |
| 12 | 1 | gi\|6680748 | 400 | 59830 | 11 | ATP synthase subunit alpha, mitochondrial precursor |
| 13 | 1 | gi\|809561 | 391 | 41335 | 11 | actin |
| 13 | 2 | gi\|74178253 | 307 | 42080 | 10 | unnamed protein product |
| 14 | 1 | gi\|5453555 | 382 | 24579 | 7 | GTP-binding nuclear protein Ran |
| 15 | 1 | gi\|6680674 | 332 | 56105 | 12 | RAC-beta serine/threonine-protein kinase (Akt2) |
| 15 | 2 | gi\|6753034 | 292 | 56071 | 13 | RAC-alpha serine/threonine-protein kinase (Akt1) |
| 15 | 3 | gi\|190883484 | 169 | 56078 | 12 | RAC-gamma serine/threonine-protein kinase (Akt3) |
| 16 | 1 | gi\|84662730 | 319 | 67573 | 10 | far upstream element-binding protein 1 |
| 16 | 2 | gi\|159163538 | 176 | 9772 | 4 | Chain A, solution structure Of Kh domain in fuse binding protein 1 |
| 17 | 1 | gi\|76779273 | 314 | 59559 | 7 | Hspd1 protein, partial |
| 18 | 1 | gi\|148706277 | 307 | 79634 | 6 | mCG140911, isoform CRA_a |
| 19 | 1 | gi\|148747424 | 298 | 33111 | 7 | ADP/ATP translocase 1 |
| 19 | 2 | gi\|22094075 | 285 | 33138 | 6 | ADP/ATP translocase 2 |
| 20 | 1 | gi\|18079339 | 297 | 86151 | 10 | aconitate hydratase, mitochondrial precursor |
| 21 | 1 | gi\|6754994 | 286 | 37987 | 5 | poly(rC)-binding protein 1 |
| 21 | 2 | gi\|148699884 | 180 | 36171 | 4 | poly(rC) binding protein 3, isoform CRA_b |
| 21 | 3 | gi\|1360003 | 145 | 37203 | 4 | nuclear poly(C)-binding protein, splicevariant E |
| 22 | 1 | gi\|468546 | 263 | 57753 | 6 | CCT (chaperonin containing TCP-1) beta subunit |
| 23 | 1 | gi\|60502437 | 256 | 49026 | 7 | protein disulfide isomerase associated 6 |
| 24 | 1 | gi\|387422 | 247 | 36044 | 6 | malate dehydrogenase |
| 25 | 1 | gi\|4759034 | 246 | 49228 | 7 | eukaryotic peptide chain release factor subunit 1 |
| 26 | 1 | gi\|33239431 | 242 | 56689 | 7 | protein RCC2 |
| 27 | 1 | gi\|200255 | 231 | 24664 | 4 | protein carboxyl methyltransferase |
| 28 | 1 | gi\|192050 | 221 | 48123 | 4 | mitochondrial aspartate aminotransferase |
| 29 | 1 | gi\|74204678 | 214 | 96138 | 10 | unnamed protein product |
| 30 | 1 | gi\|20809354 | 205 | 59422 | 6 | polypyrimidine tract binding protein 1 |
| 31 | 1 | gi\|6753320 | 198 | 61162 | 8 | T-complex protein 1 subunit gamma |
| 32 | 1 | gi\|6754632 | 196 | 41648 | 6 | mitogen-activated protein kinase 1 (ERK1) |
| 33 | 1 | gi\|74195705 | 193 | 51272 | 5 | unnamed protein product |
| 34 | 1 | gi\|31982332 | 192 | 42834 | 5 | glutamine synthetase |
| 35 | 1 | gi\|70794816 | 189 | 47453 | 9 | uncharacterized protein LOC433182 |
| 36 | 1 | gi\|16716569 | 183 | 26802 | 1 | protease, serine, 1 precursor |
| 37 | 1 | gi\|12846904 | 177 | 32954 | 4 | unnamed protein product |
| 38 | 1 | gi\|74151643 | 176 | 60159 | 5 | unnamed protein product |
| 39 | 1 | gi\|26353794 | 170 | 57103 | 11 | unnamed protein product |
| 40 | 1 | gi\|6754524 | 160 | 36817 | 6 | L-lactate dehydrogenase A chain isoform 1 |
| 41 | 1 | gi\|387397 | 160 | 57978 | 4 | epidermal keratin subunit I, partial |
| 41 | 2 | gi\|148670626 | 105 | 55010 | 3 | mCG144006 |
| 42 | 1 | gi\|110625979 | 151 | 50371 | 3 | elongation factor 1-gamma |
| 43 | 1 | gi\|6753086 | 144 | 35867 | 3 | DNA-(apurinic or apyrimidinic site) lyase |
| 44 | 1 | gi\|1705525 | 140 | 83145 | 3 | RecName: Full=DNA replication licensing factor MCM5 |
| 45 | 1 | gi\|8895708 | 136 | 43281 | 3 | DAZ-associated protein 1 |
| 46 | 1 | gi\|124028629 | 135 | 37437 | 2 | RecName: Full=heterogeneous nuclear ribonucleoproteins A2/B1 |
| 47 | 1 | gi\|27370092 | 135 | 49876 | 2 | elongation factor Tu, mitochondrial isoform 1 |
| 48 | 1 | gi\|50814 | 134 | 44692 | 5 | unnamed protein product |
| 49 | 1 | gi\|201725 | 132 | 57952 | 5 | t complex polypeptide 1 |
| 50 | 1 | gi\|6753322 | 128 | 58543 | 5 | T-complex protein 1 subunit delta |
| 50 | 2 | gi\|37359776 | 71 | 60143 | 2 | mKIAA0098 protein |
| 51 | 1 | gi\|12851716 | 127 | 35146 | 2 | unnamed protein product |
| 52 | 1 | gi\|74180977 | 125 | 235459 | 7 | unnamed protein product |
| 53 | 1 | gi\|26345686 | 125 | 57344 | 2 | unnamed protein product |
| 54 | 1 | gi\|12859782 | 121 | 66099 | 3 | unnamed protein product |
| 54 | 2 | gi\|148672085 | 80 | 38347 | 2 | mCG144996 |
| 54 | 3 | gi\|148672069 | 69 | 59516 | 2 | cDNA sequence BC031593 |
| 54 | 4 | gi\|293686 | 42 | 59756 | 2 | epidermal keratin subunit II |
| 55 | 1 | gi\|14250204 | 119 | 101876 | 5 | Methylenetetrahydrofolate dehydrogenase (NADP+ dependent) |
| 56 | 1 | gi\|21704096 | 117 | 44918 | 2 | TAR DNA-binding protein 43 isoform 1 |
| 57 | 1 | gi\|1083269 | 117 | 59890 | 6 | CW17R protein - mouse |
| 58 | 1 | gi\|10946928 | 116 | 49454 | 3 | heterogeneous nuclear ribonucleoprotein H |
| 58 | 2 | gi\|148670393 | 113 | 37396 | 3 | mCG50680 |
| 59 | 1 | gi\|116283229 | 111 | 41824 | 5 | proliferation-associated protein 2G4 (EBP1) |
| 60 | 1 | gi\|488513 | 108 | 68661 | 3 | EWS |
| 61 | 1 | gi\|74151988 | 106 | 58390 | 4 | unnamed protein product |
| 62 | 1 | gi\|12851415 | 105 | 29963 | 3 | unnamed protein product |
| 63 | 1 | gi\|148685496 | 103 | 43881 | 2 | mCG22383, isoform CRA_c |
| 64 | 1 | gi\|21450129 | 102 | 45129 | 3 | acetyl-CoA acetyltransferase, mitochondrial precursor |
| 65 | 1 | gi\|6677775 | 100 | 14807 | 2 | 60S ribosomal protein L22 |
| 66 | 1 | gi\|30749336 | 99 | 18975 | 3 | crystal structure of Gga1 Gat N-terminal region in complex with Arf1 Gtp form |
| 67 | 1 | gi\|18204423 | 98 | 64751 | 3 | Picalm protein |
| 68 | 1 | gi\|6755372 | 98 | 26828 | 1 | 40S ribosomal protein S3 |
| 69 | 1 | gi\|6680229 | 98 | 24318 | 4 | high mobility group protein B2 |
| 69 | 2 | gi\|148673204 | 65 | 25952 | 4 | mCG5336 |
| 70 | 1 | gi\|6678359 | 93 | 68272 | 3 | transketolase |
| 71 | 1 | gi\|7242156 | 91 | 25120 | 2 | acyl-protein thioesterase 2 |
| 72 | 1 | gi\|21450325 | 89 | 22297 | 2 | flavin reductase (NADPH) |
| 73 | 1 | gi\|119850791 | 88 | 55424 | 2 | Krt78 protein |
| 74 | 1 | gi\|74142919 | 86 | 239074 | 1 | unnamed protein product |
| 75 | 1 | gi\|26344461 | 86 | 22991 | 3 | unnamed protein product |
| 76 | 1 | gi\|199025 | 85 | 118230 | 2 | microtubule-associated protein 4 |
| 77 | 1 | gi\|90787451 | 85 | 120743 | 2 | putative gag-pol protein |
| 78 | 1 | gi\|74204693 | 84 | 102651 | 3 | unnamed protein product |
| 79 | 1 | gi\|74142035 | 84 | 113492 | 2 | unnamed protein product |
| 80 | 1 | gi\|6754222 | 84 | 30926 | 1 | heterogeneous nuclear ribonucleoprotein A/B isoform 2 |
| 81 | 1 | gi\|3550456 | 84 | 96520 | 2 | Alix |
| 82 | 1 | gi\|74205649 | 84 | 56835 | 1 | unnamed protein product |
| 83 | 1 | gi\|35505176 | 83 | 99704 | 3 | Cand1 protein, partial |
| 84 | 1 | gi\|111308159 | 83 | 71336 | 2 | keratin 2 |
| 85 | 1 | gi\|4504445 | 82 | 34289 | 1 | heterogeneous nuclear ribonucleoprotein A1 isoform a |
| 86 | 1 | gi\|26345208 | 81 | 64740 | 1 | unnamed protein product |
| 87 | 1 | gi\|1167510 | 80 | 14281 | 2 | TI-225 |
| 88 | 1 | gi\|3065929 | 79 | 28516 | 1 | 14-3-3 protein gamma |
| 89 | 1 | gi\|9963901 | 78 | 16056 | 1 | profilin II |
| 90 | 1 | gi\|11230802 | 77 | 105368 | 2 | alpha-actinin |
| 91 | 1 | gi\|191765 | 76 | 48792 | 1 | alpha-fetoprotein, partial |
| 92 | 1 | gi\|9483736 | 75 | 35296 | 4 | succinyl-CoA synthetase |
| 93 | 1 | gi\|18044175 | 74 | 56716 | 1 | Hyou1 protein |
| 94 | 1 | gi\|74219852 | 73 | 77152 | 1 | unnamed protein product |
| 95 | 1 | gi\|545439 | 73 | 2112 | 1 | Erp61, GRP58=stress-inducible luminal endoplasmic reticulum protein |
| 96 | 1 | gi\|26332671 | 73 | 106504 | 3 | unnamed protein product |
| 97 | 1 | gi\|5020213 | 71 | 37360 | 3 | mitotic checkpoint protein BUB3 |
| 98 | 1 | gi\|1666689 | 70 | 221379 | 1 | alpha-NAC, muscle-specific form gp220 |
| 99 | 1 | gi\|74209418 | 69 | 72338 | 2 | unnamed protein product |
| 100 | 1 | gi\|19526912 | 67 | 41801 | 3 | hsc70-interacting protein |
| 101 | 1 | gi\|31127291 | 67 | 64015 | 2 | Rangap1 protein |
| 102 | 1 | gi\|49257190 | 66 | 31952 | 1 | enoyl coenzyme A hydratase, short chain, 1, mitochondrial |
| 103 | 1 | gi\|6754910 | 66 | 38334 | 2 | nuclear migration protein nudc |
| 104 | 1 | gi\|149251776 | 66 | 32817 | 2 | PREDICTED: nucleophosmin-like |
| 105 | 1 | gi\|148708897 | 65 | 24594 | 1 | peroxiredoxin 4, isoform CRA_b |
| 106 | 1 | gi\|6756041 | 65 | 27925 | 2 | 14-3-3 protein zeta/delta |
| 107 | 1 | gi\|28077013 | 64 | 47972 | 1 | stromal membrane-associated protein 1 |
| 108 | 1 | gi\|23271826 | 63 | 112497 | 2 | ubiquitin associated protein 2-like |
| 109 | 1 | gi\|9790077 | 63 | 47194 | 2 | glycogen synthase kinase-3 beta |
| 110 | 1 | gi\|66571305 | 63 | 64410 | 1 | nuclear RNA export factor 3 |
| 111 | 1 | gi\|74202572 | 61 | 50385 | 2 | unnamed protein product |
| 112 | 1 | gi\|33468931 | 61 | 61641 | 1 | serine--tRNA ligase, cytoplasmic isoform 1 |
| 113 | 1 | gi\|124001574 | 60 | 112708 | 2 | general transcription factor II-I isoform 1 (TFII-I) |
| 114 | 1 | gi\|12834035 | 59 | 31898 | 1 | unnamed protein product |
| 115 | 1 | gi\|22094123 | 59 | 120988 | 1 | transcription elongation factor SPT5 |
| 116 | 1 | gi\|22094989 | 58 | 39980 | 1 | mitochondrial import inner membrane translocase subunit TIM50 precursor |
| 117 | 1 | gi\|2495342 | 58 | 94872 | 2 | RecName: Full=heat shock 70 kDa protein 4 |
| 118 | 1 | gi\|3097244 | 58 | 16462 | 1 | ribosomal protein S14 |
| 119 | 1 | gi\|4519256 | 57 | 56550 | 1 | 4F2/CD98 light chain |
| 120 | 1 | gi\|387106 | 56 | 46488 | 1 | aspartate aminotransferase |
| 121 | 1 | gi\|113680348 | 50 | 55215 | 2 | fascin |
| 122 | 1 | gi\|74204023 | 56 | 113830 | 3 | unnamed protein product |
| 123 | 1 | gi\|74203154 | 55 | 87775 | 1 | unnamed protein product |
| 124 | 1 | gi\|7305075 | 54 | 51854 | 1 | ras GTPase-activating protein-binding protein 1 |
| 125 | 1 | gi\|6755963 | 54 | 30851 | 1 | voltage-dependent anion-selective channel protein 1 |
| 126 | 1 | gi\|471976 | 53 | 39221 | 2 | protein phosphatase 1 (PP-1) |
| 127 | 1 | gi\|200065 | 53 | 62956 | 1 | neuroleukin |
| 128 | 1 | gi\|55217 | 51 | 89936 | 3 | valosin-containing protein |
| 129 | 1 | gi\|12848861 | 51 | 31398 | 1 | unnamed protein product |
| 130 | 1 | gi\|555823 | 51 | 58283 | 1 | pendulin |
| 131 | 1 | gi\|13385942 | 47 | 51988 | 3 | citrate synthase, mitochondrial precursor |
| 132 | 1 | gi\|6755893 | 50 | 26941 | 1 | trypsin 4 precursor |
| 133 | 1 | gi\|50510319 | 50 | 60817 | 2 | mKIAA0002 protein |
| 134 | 1 | gi\|12836608 | 50 | 47968 | 1 | unnamed protein product |
| 135 | 1 | gi\|126723336 | 49 | 33276 | 1 | prohibitin-2 |
| 136 | 1 | gi\|9506571 | 49 | 36371 | 1 | eukaryotic translation initiation factor 2 subunit 1 |
| 137 | 1 | gi\|21703842 | 49 | 55727 | 3 | tRNA-splicing ligase RtcB homolog |
| 138 | 1 | gi\|19527026 | 48 | 35312 | 1 | leucine-rich repeat-containing protein 59 |
| 139 | 1 | gi\|26389719 | 48 | 86946 | 1 | unnamed protein product |
| 140 | 1 | gi\|13096978 | 48 | 72511 | 2 | Poly(A) binding protein, cytoplasmic 4 |
| 141 | 1 | gi\|26346400 | 47 | 57567 | 2 | unnamed protein product |
| 142 | 1 | gi\|12846591 | 47 | 33782 | 2 | unnamed protein product |
| 143 | 1 | gi\|19526940 | 46 | 58714 | 1 | 60S ribosomal export protein NMD3 |
| 144 | 1 | gi\|12836885 | 46 | 123917 | 1 | nonsense mRNA reducing factor 1 NORF1 |
| 145 | 1 | gi\|4506413 | 46 | 21316 | 1 | ras-related protein Rap-1A |
| 146 | 1 | gi\|74228628 | 45 | 67719 | 1 | unnamed protein product |
| 147 | 1 | gi\|6753324 | 44 | 58424 | 1 | T-complex protein 1 subunit zeta |
| 148 | 1 | gi\|124517678 | 44 | 185956 | 1 | tyrosine-protein phosphatase non-receptor type 23 |
| 149 | 1 | gi\|6679803 | 43 | 11972 | 1 | peptidyl-prolyl cis-trans isomerase FKBP1A |
| 150 | 1 | gi\|66267550 | 43 | 168127 | 1 | Eprs protein, partial |
| 151 | 1 | gi\|23956214 | 43 | 75508 | 1 | splicing factor, proline- and glutamine-rich |
| 152 | 1 | gi\|12860337 | 42 | 32527 | 1 | unnamed protein product |
| 153 | 1 | gi\|4590328 | 42 | 141523 | 1 | valyl-tRNA synthetase |
| 154 | 1 | gi\|12848061 | 42 | 97389 | 1 | unnamed protein product |
| 155 | 1 | gi\|14587839 | 42 | 37930 | 1 | acyl-CoA hydrolase |
| 156 | 1 | gi\|19526818 | 42 | 40063 | 1 | phosphate carrier protein, mitochondrial precursor |
| 159 | 1 | gi\|13435984 | 41 | 56239 | 1 | serine hydroxymethyltransferase 2 (mitochondrial) |
| 160 | 1 | gi\|885932 | 40 | 21963 | 1 | peroxidase |
| 161 | 1 | gi\|22122515 | 40 | 38321 | 1 | activator of 90 kDa heat shock protein ATPase homolog 1 |
| 162 | 1 | gi\|91171 | 39 | 50850 | 1 | peripherin (clone 3u) - mouse (fragment) |
| 163 | 1 | gi\|26345546 | 39 | 59567 | 1 | unnamed protein product |
| 164 | 1 | gi\|12861019 | 38 | 21485 | 1 | unnamed protein product |
| 165 | 1 | gi\|148685432 | 37 | 101939 | 2 | ataxin 2-like, isoform CRA_a |
| 166 | 1 | gi\|31560731 | 37 | 68625 | 1 | V-type proton ATPase catalytic subunit A |
| 167 | 1 | gi\|12963511 | 37 | 16076 | 1 | 40S ribosomal protein S19 |
| 168 | 1 | gi\|23956082 | 37 | 34607 | 1 | 60S ribosomal protein L5 |
| 169 | 1 | gi\|26332465 | 36 | 37516 | 1 | unnamed protein product |
| 170 | 1 | gi\|53000 | 36 | 271556 | 1 | microtubule-associated protein 1B |
| 171 | 1 | gi\|13096878 | 35 | 86574 | 1 | HGF-regulated tyrosine kinase substrate |
| 172 | 1 | gi\|4506605 | 34 | 14970 | 1 | 60S ribosomal protein L23 |
| 173 | 1 | gi\|3851614 | 34 | 59249 | 1 | succinate dehydrogenase Fp subunit |
| 174 | 1 | gi\|6756051 | 33 | 49719 | 1 | zinc finger protein 207 isoform 4 |
| 175 | 1 | gi\|4503545 | 32 | 17049 | 1 | eukaryotic translation initiation factor 5A-1 isoform B |
| 176 | 1 | gi\|60360164 | 32 | 135859 | 1 | mKIAA0886 protein |
| 177 | 1 | gi\|109866 | 31 | 40016 | 1 | gene DN38 protein - mouse (fragment) |
| 178 | 1 | gi\|26325850 | 30 | 68656 | 1 | unnamed protein product |
| 179 | 1 | gi\|37360414 | 30 | 74678 | 1 | mKIAA1499 protein |

Proteins highlighted were not found in the negative control samples and reproduced in three independent experiments. These proteins were identified as Akt binding partners.
